# Tree-Inspired Structurally Graded Aerogel with Synergistic Water, Salt, and Thermal Transport for High-Salinity Solar-Powered Evaporation

**DOI:** 10.1007/s40820-024-01448-8

**Published:** 2024-06-17

**Authors:** Xiaomeng Zhao, Heng Zhang, Kit-Ying Chan, Xinyue Huang, Yunfei Yang, Xi Shen

**Affiliations:** 1 Department of Aeronautical and Aviation Engineering, The Hong Kong Polytechnic University, Hung Hom, Kowloon, Hong Kong SAR People’s Republic of China; 2 Research Institute for Sports Science and Technology, The Hong Kong Polytechnic University, Hung Hom, Kowloon, Hong Kong SAR People’s Republic of China; 3 Research Institute for Advanced Manufacturing, The Hong Kong Polytechnic University, Hung Hom, Kowloon, Hong Kong SAR People’s Republic of China

**Keywords:** Composite aerogel, Graded structure, Solar-powered evaporation, Thermal insulation, Salt rejection

## Abstract

Solar-powered interfacial evaporation is an energy-efficient solution for water scarcity. It requires solar absorbers to facilitate upward water transport and limit the heat to the surface for efficient evaporation. Furthermore, downward salt ion transport is also desired to prevent salt accumulation. However, achieving simultaneously fast water uptake, downward salt transport, and heat localization is challenging due to highly coupled water, mass, and thermal transport. Here, we develop a structurally graded aerogel inspired by tree transport systems to collectively optimize water, salt, and thermal transport. The arched aerogel features root-like, fan-shaped microchannels for rapid water uptake and downward salt diffusion, and horizontally aligned pores near the surface for heat localization through maximizing solar absorption and minimizing conductive heat loss. These structural characteristics gave rise to consistent evaporation rates of 2.09 kg m^−2^ h^−1^ under one-sun illumination in a 3.5 wt% NaCl solution for 7 days without degradation. Even in a high-salinity solution of 20 wt% NaCl, the evaporation rates maintained stable at 1.94 kg m^−2^ h^−1^ for 8 h without salt crystal formation. This work offers a novel microstructural design to address the complex interplay of water, salt, and thermal transport.

**Supplementary Information:**

The online version contains supplementary material available at 10.1007/s40820-024-01448-8.

## Highlights

- Inspired by transport system in trees, a two-way water and salt transport mechanism is realized in a structurally graded aerogel, enabling simultaneous fast water uptake and salt rejection.
- The horizontally aligned pore channels near the surface achieve excellent heat localization by maximizing solar absorption and minimizing heat loss.
- The integrated water, salt, and thermal transports impart an impressive evaporation rate of 1.94 kg m^−2^ h^−1^ in a 20 wt% NaCl solution for 8 h without salt accumulation.

**Supplementary Information:**

The online version contains supplementary material available at 10.1007/s40820-024-01448-8.

## Graphical abstract

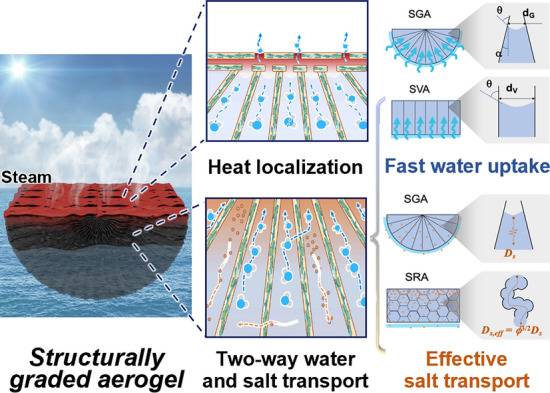

## Introduction

Water scarcity has become an urgent global challenge requiring sustainable and environmentally friendly solutions [1–3]. Solar-powered evaporation, leveraging solar energy to evaporate seawater or wastewater for conversion into clean and potable water, is a highly promising technology because of its energy-saving potential with renewable solar energy as the sole energy source [4]. This technology relies on solar absorbers to capture incident sunlight and convert it into highly concentrated thermal energy confined at the water–air interface where highly efficient water evaporation occurs [5]. Three-dimensional (3D) solar absorbers in the form of fabrics [6, 7], aerogels [8–11], and hydrogels [12–14] have been developed from a range of photothermal materials such as carbon nanotubes (CNTs), graphene, and MXene. These materials have been designed as standalone evaporators serving on water surfaces to enable solar absorption, water transport, and heat localization through engineering their highly porous structures, contributing to high solar-to-vapor energy conversion efficiency of over 90% and evaporation rates close to the theoretical limit (~ 1.47 kg m^−2^ h^−1^) under standard one-sun illumination, promising for high-yield desalination and wastewater treatment applications [12]. Despite these promising properties, one major drawback of 3D solar absorbers is the salt accumulation especially under high-salinity situations. During evaporation of high-salinity brine or high-concentration wastewater, salts, minerals, and impurities precipitated from the solution and accumulated on the evaporation surface due to supersaturation of the surface solution [15, 16], reducing the solar absorption and impeding the water transport which in turn reduced the overall evaporation performance [17–19]. To avoid salt accumulation, smart structural designs such as evaporators with dual evaporation modes have been proposed to redissolve the salts by self-rotating [20]. Although accumulated salts could be removed by subsequent flushing [21], dissolved by self-cleaning [22], or collected by gravity-assisted methods [23], these time-consuming steps were deleterious to continuous operations. Localizing salts on evaporation surface is another effective strategy, which can be realized by controlling the brine transport or non-contact design [24], leading to zero liquid discharge during the evaporation process [25]. While non-contact evaporators [26–28] avoided salt crystal formation on their surfaces because of the physical separation from seawater, such separation in turn gave rise to low energy conversion efficiency and thus relatively low evaporation rates (~ 1.3 kg m^−2^ h^−1^). Circumventing salt accumulation on 3D evaporators without impairing their rapid water transport and heat localization remains a significant challenge for practical desalination under high-salinity conditions, primarily due to highly coupled water, salt, and thermal transport in the evaporators.

Several strategies have been developed to tackle the salt accumulation issue through decoupling water, salt, and thermal transports, including Janus surface design [29, 30], ion rejection [17, 31, 32], salt localization [23, 33], and diffusive/convective backflow [12, 34–37]. The Janus design stacked hydrophobic photothermal layer and hydrophilic water absorbing layer in tandem to prevent brine transport onto the photothermal surface. Nevertheless, the separating functions of water transport and solar absorption discourage fast water transport to the surface, not ideal for a high evaporation rate. Salt localization involved the directional transport of brine to a confined location for crystallization. The heat loss associated with brine transport was rather significant, undesirable for a high energy efficiency. Ion rejection encompassed the incorporation of ion-selective groups or species in hydrogels to manipulate the transport of salt ions through ionic interactions. Although salt crystal formation was completely eliminated through the ion rejection mechanism, water transport channels, solar absorption layers, and thermal insulation design were implemented separately to allow effective evaporation. All in all, these approaches decoupled thermal, salt, and water transports by integrating different functional constituents to achieve simultaneous heat localization, fast water delivery, and salt rejection, but inevitably complicated the whole system because of separated components for individual functions.

Compared to the above, diffusive/convective backflow is a promising strategy to use a single structure for salt rejection without compromising heat localization and water transport. The principle involved diffusive or convective flows driven by salt concentration gradient to prevent surface solution from reaching saturation [38]. Proper structural design is essential to ensure fast downward salt ion diffusion or convection, avoiding salt crystal precipitation at the heating surface [19]. Commonly, solar absorbers have been engineered with vertically aligned pores using techniques such as freeze-casting [39], serving as direct conduits for water uptake and fast salt ion diffusion [12, 35, 36]. The diffusion could be further enhanced by using macroscopic channels [40, 41]. For example, through-the-thickness channels of millimeter-scale diameters were drilled into a wood panel, inducing salt ion transport from micron-size xylem channels to millimeter-size channels for enhanced salt rejection [41]. However, the macroscopic channels inadvertently accelerated heat conduction through water and vertical pore walls, resulting in increased heat loss and reduced energy efficiency [42, 43]. Further tuning the dimensions of macroscopic channels led to engineered fluidic flow with passive convection driven by salinity gradients [44]. Leveraging the substantial disparity between the low salt diffusivity in water and the high thermal diffusivity of water, an optimized channel size was attained to enable salt rejection through convective flow while maintaining the heat transport in a diffusive regime to minimize heat loss, ultimately achieving a high efficiency of over 80% in a high-salinity solution of 20 wt%.

Despite these ameliorating efforts in balancing salt and thermal transport in vertical pores, the energy efficiency and evaporation rate are still not satisfactory due to inevitable downward thermal transport through solid pore walls and high-thermal-conductivity water. To reduce the downward thermal conduction, horizontal pores were preferred over vertical ones because of the low thermal conductivity (TC) across the pore alignment direction [45–47]. This conflicting requirement makes it challenging to achieve excellent thermal localization concurrently with water transport and salt rejection, necessitating complex bidirectional structural features to reach a tradeoff. Attempt has been made to develop a feather-like anisotropic hierarchical structure with longitudinal struts and transverse ligaments [48], efficiently delivering water to the evaporating surface while providing exceptional transverse insulation. A vertical radiant structure was also designed to allow salt diffusion through vertical pores at the center while reducing the heat conduction through radially arranged horizontal vessels, striking a balance between thermal localization and salt resistance [49]. While the bidirectional pores allow fast downward salt diffusion and good insulation, the upward water transport was not optimized simultaneously. It is challenging to realize fast upward water transport for evaporation and downward ion transport for salt rejection at the same time while also maintaining good thermal insulation for high energy efficiency because of highly coupled water, salt, and thermal transport.

**Fig. 1:**
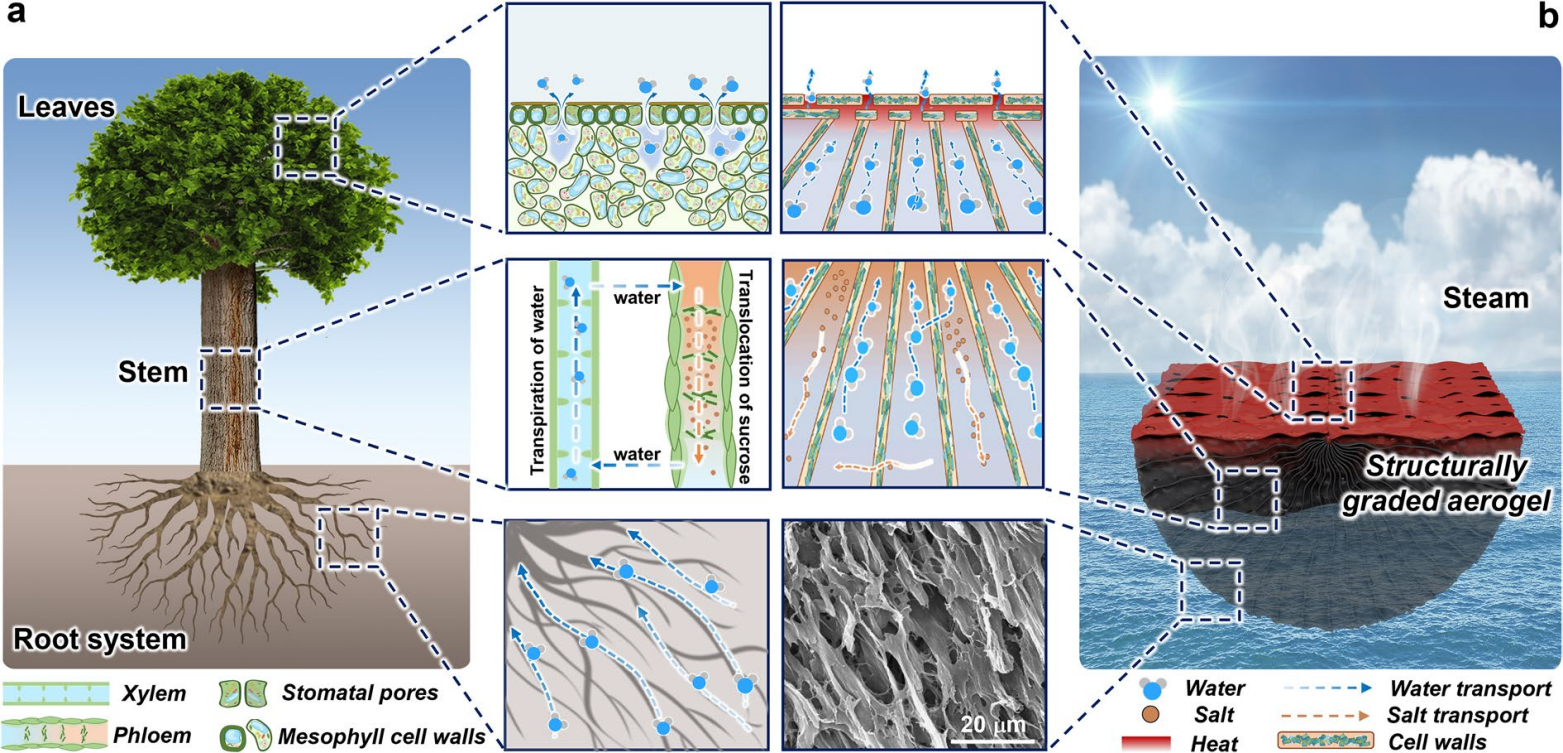
Biomimetic design of SGA. Schematics showing the water and mass transport mechanisms in **a** trees and **b** SGA

In this study, we propose an integrated strategy to synergistically optimize water, salt, and thermal transport through developing a structurally graded aerogel (SGA) for two-way water and salt transport as well as heat localization. The multiscale structures of SGA were designed to mimic the entire transport system of trees (Fig. 1). The fan-shaped, tapered channels delivered a two-way water and salt transport mechanism for fast water uptake and downward salt diffusion. Simultaneously, the horizontally aligned surface pore channels achieved excellent heat localization through maximizing the solar absorption and minimizing thermal emission and conduction. The co-optimization of water, salt, and heat transport gave rise to consistent evaporation rates averaging at 2.09 kg m^−2^ h^−1^ under one-sun illumination in a 3.5 wt% NaCl solution for 7 days without degradation. Even for a high-salinity solution of 20 wt% NaCl, the evaporation rates maintained stable at 1.94 kg m^−2^ h^−1^ for 8 h without salt crystal formation. These results underscore the significant potential of SGA for solar-powered desalination and purification of high-salinity brines or high-concentration wastewaters.

## Experimental Section

### Materials

Polyvinyl alcohol (PVA, 99 + % hydrolyzed, molecular weight of 146,000 to 186,000) was supplied by Sigma-Aldrich. Graphene oxide (GO) solution (2 mg mL^−1^) and CNTs (> 95%, carboxyl content: 3.86%) were purchased from XFNANO, China. Sodium chloride (NaCl, 99.5%) was supplied by Aladdin Chemistry Co. Ltd. All the materials and chemicals were used without any treatment. Seawater was taken from the Victoria Harbour of South China Sea near the Hong Kong Polytechnic University.

### Fabrication of SGA

**Fig. 2:**
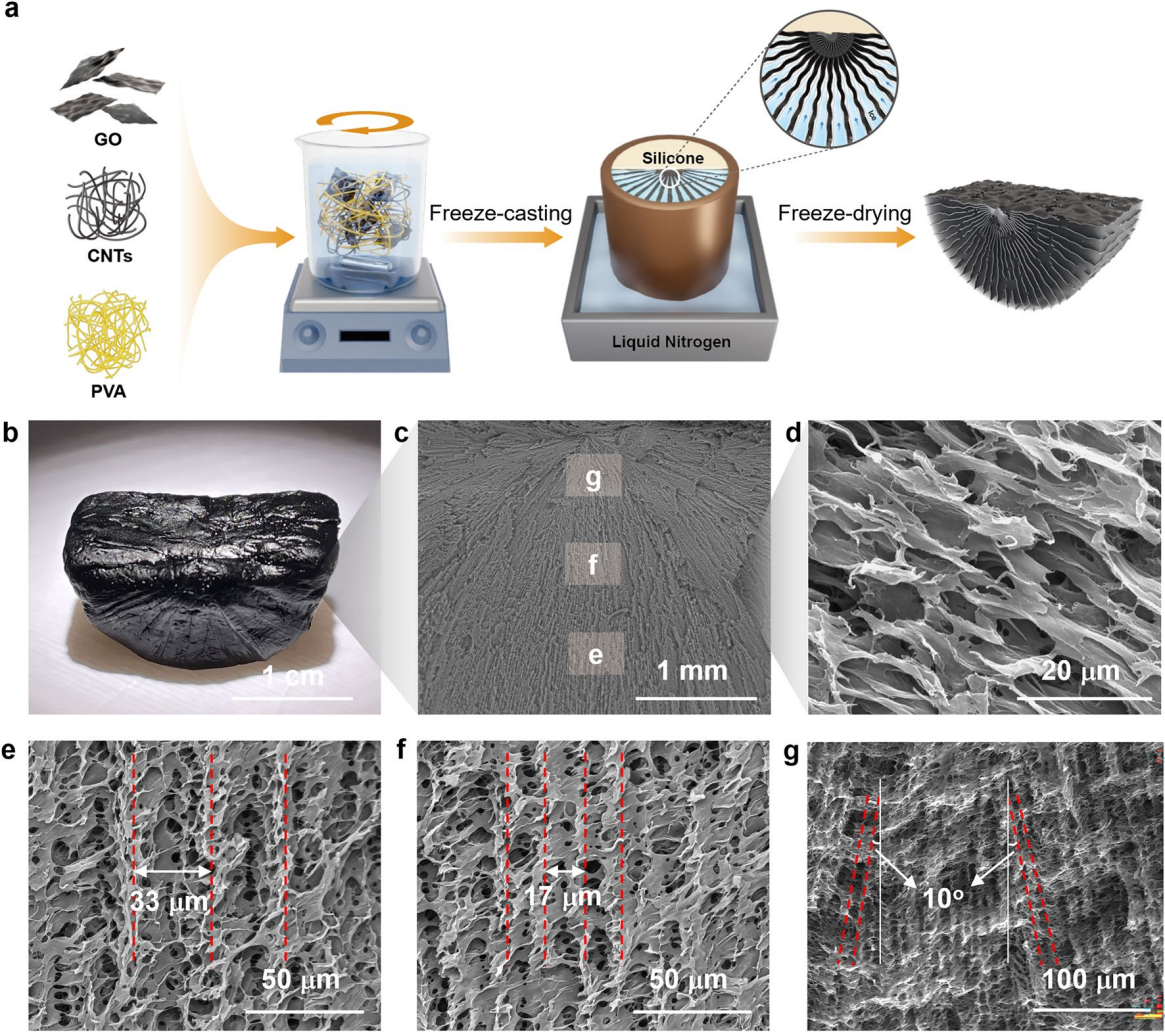
Fabrication and structural characteristics of SGA. **a** Schematic of fabrication of SGA with fan-shaped gradient structures. **b** Photograph of SGA showing its black appearance and hull-like arched bottom. SEM images showing **c** the fan-like cell walls and **d** fine-root-like secondary ligaments of SGA. **e–g** High-magnification SEM images showing detailed channel widths at different positions marked in (**c**)

First, 10 g PVA powders were dissolved in 100 g deionized (DI) water at 130 °C for 2 h. Then, the GO solution and CNT powders were mixed at a ratio of 3:7 and stirred continuously for 3 h followed by ultrasonication for 0.5 h at room temperature until a uniform GO-CNT dispersion was obtained. Subsequently, the required amount of PVA solution was added to the above GO-CNT dispersion, and the mixture was continuously stirred at room temperature for 3 h to obtain a GO-CNT/PVA mixture solution. SGA was fabricated by radial freeze-casting followed by freeze-drying (as shown in Fig. 2a later). The GO-CNT/PVA mixture solution was poured into a copper mold which was partially immersed in liquid nitrogen bath. After the solution was frozen completely, the sample was freeze-dried in a freeze-dryer (Scientz-10n) at 5 Pa for 36 h. For comparison, structurally vertical aerogel (SVA) and structurally random aerogel (SRA) were also prepared with the same steps except that GO-CNT/PVA mixture solution was freeze-cast vertically using a bottom copper cold source and randomly in a refrigerator, respectively.

### Characterization

The sample morphologies and microstructures were observed using the scanning electron microscopy (SEM, Tescan VEGA3). The solar absorption spectra were measured using a ultraviolet–visible-near infrared (UV–vis-NIR) spectrophotometer (3700DUV, Shimadzu) from 250 to 2500 nm with an integrating sphere. The solar absorption (A) was calculated by A = 1-R-T, where R and T are the reflection and transmission, respectively. The TCs of different aerogels were measured based on a transient plane heat source method (Hot Disk TPS2500S). The IR images were taken using an IR camera (FLIR-B256V, Guide Sensmart). Raman spectroscopy (InVia, Renishaw, 514 nm laser) was used to identify the presence of carbon nanomaterials in the aerogels. The real-time temperature distributions were measured using thermocouples and recorded by a data logger (CENTER 309). Ion concentrations were measured using an inductively coupled plasma optical emission spectrometer (ICP-OES, ﻿Agilent 5110). Tensile and compressive properties were measured on a Shimadzu AGS-X Tester.

### Solar-Powered Water Evaporation Experiments

To systematically compare the evaporation performance of aerogels with different structures, a series of water evaporation tests were conducted at a relative humidity of 50% and ambient temperature. The aerogel sample was floated on the water surface in a glass beaker with the surrounding water being covered with a polyethylene foam to avoid the direct evaporation of bulk water. The sample was continuously illuminated by a solar simulator (CEL-S500/350) for 1 h under various solar intensities measured by a solar power meter (TES-1333R). The mass loss of water was recorded in real time by an electronic balance (FA2004, 0.1 mg). At least two independent measurements were carried out for each test.

### Salinity Measurements

The salinity was measured using a digital refractometer (DLX-ARHT028). A small amount of brine was carefully collected from the water–air interface or the bulk brine using a glass point capillary tube with an inner diameter of 0.9 mm and length of 100 mm. The collected brine was dripped onto the lens of the digital refractometer for reading the salinity.

## Results and Discussion

### Biomimetic Design of SGA

Trees are natural solar-driven evaporators capable of passively transporting water from roots to leaves via their stems [50]. Tree roots play a dominant role in absorbing water from the soil, which is then transported up through stems to leaves for photosynthesis. Stems are also responsible for transferring sugars made in the leaves during photosynthesis downwards to other parts of the tree. Meanwhile, the water evaporates from leaves through cell wall pores under sunlight, driving the continuous water uptake through capillary forces. The spontaneous, solar-driven mass transport can be attributed to several unique structural features in roots, stems and leaves, as shown in Fig. 1a [45]. First, roots grow radially in all directions to maximize the contact area and thus accessible water in the soil. Moreover, fine roots at the tips are covered by root hairs to increase the surface area for water absorption. Second, the absorbed water is passively transported upwards through the xylem driven by the water pressure gradient owing to water evaporation from the leaves. Meanwhile, sugars produced from leaves create a concentration gradient in the phloem, driving the downward transportation of sugars from leaves to roots by diffusion. Third, leaves with large surface areas are capable of absorbing sunlight for transpiration, where water evaporates from the mesophyll cell walls through the stomatal pores on the leaf surfaces, increasing the surface tension on the water menisci and thus pumping the water up in the xylem channels. While evaporators with vertical channels mimicking the xylem vessels for water transport have been well developed [41, 51–53], little effort has been made to fully imitate the entire structures and mechanisms of trees for synergistic upward water transport and downward salt transport for simultaneous water evaporation and salt rejection. Here, we designed and fabricated an SGA to mimic the intricate transport system in trees (Fig. 1b). The SGA was made from a mixture of GO, CNTs, and PVA to endow both excellent sunlight absorption and hydrophilicity [54]. The SGA had a boat-like, arched bottom with fan-shaped walls radiating from the top to bottom, resembling the tree root structure which gave rise to a better stability and larger water contact area than a flat surface for water absorption. Similar to fine roots, the whisker-like struts grown from the main cell walls in SGA further improved the surface area for water absorption. Meanwhile, the pore channels formed between radiating cell walls tapered down to the evaporating surface, facilitating fast upward water transport driven by water potential gradient and downward salt diffusion induced by salt concentration gradient, mimicking the two-way water and mass transport mechanism in the xylem and phloem. Furthermore, the flat evaporating surface formed by horizontal cell walls served the same function as leaves, providing large surface areas for sunlight absorption and numerous pores for vapor escape. The horizontally aligned pores near the surface also limited the downward heat conduction [55, 56], beneficial to a low heat loss for high energy efficiency.

### Fabrication and Structural Characteristics of SGA

To construct the fan-shaped microstructure, the SGA was fabricated from a mixture of GO, CNT and PVA solution by a freeze-casting technique using a custom-made hollow cylindrical copper mold as the cold source (Fig. 2a). Half of the mold and the bottom were filled with low-thermal-conductivity polydimethylsiloxane (PDMS), generating a temperature gradient from peripherals to the center to force the radial growth of ice crystals (Fig. S1, detailed fabrication can be found in Experimental Section) [57, 58]. The freeze-casting technique could produce SGA of different sizes by using molds of different sizes (Fig. S2a). It was also adaptable to fabricate aerogels with other shapes such as that with a hemispherical bottom by using a half-spherical mold (Fig. S3a). The concentrations of carbon nanofillers and PVA were first optimized by changing their weight ratios and an optimized ratio of 0.05 was used in the subsequent discussion (see Supplementary Note S1 and Figs. S4 to S6 for details). Figure 2b–g shows the multiscale structural features of SGA. The SGA appeared black thanks to the presence of GO and CNT fillers as confirmed by the Raman spectra (Fig. S7), beneficial for sunlight absorption. Unlike the rectangular shape in most floating evaporators, the SGA featured a cylindrical bottom resembling the hull of a boat (Fig. 2b), essential to buoyant stability in practical operations (Fig. S8). SGA with different sizes also showed good buoyant stability (Fig. S2b). Although the hemispherical aerogel could be floated more stable than SGA because of a higher degree of stability in any in-plane directions, its evaporation performance was inferior to SGA (Fig. S3b). The SEM image of SGA (Fig. 2c) indicates fan-shaped cell walls radiating from top to peripheries because of directional ice crystal growths, much like the spreading roots to enhance the accessible area for absorbing water. Moreover, numerous secondary ligaments were observed in addition to the main cell walls (Fig. 2d), functioning the same as fine roots to increase the surface area for water absorption.

At higher magnifications, the widths of the channels between radiating cell walls gradually tapered from ~ 33 µm (Fig. 2e) at the bottom to ~ 17 µm (Fig. 2f) near the center, ultimately narrowing down to only ~ 9 µm at the top (Fig. 2g). These gradient channels imparted a high capillary pressure at the water–air interface and a salt concentration gradient from top to bottom, facilitating upward water and downward salt transport mechanism similar to that of xylem and phloem. The pore channels near the surface aligned horizontally, giving rise to a rather flat top surface (Fig. S9a). These in-plane oriented pores could contribute to reduced thermal conduction in the thickness direction, while the flat top surface increased the effective surface area for sunlight absorption compared to the vertical ones, functioning similarly to the large-area leaves for transpiration. It is noted that micro-sized pores also exist on these surface cell walls (Fig. S9b), facilitating vapor escape during evaporation. These multiscale structural characteristics mimicking the whole structures of trees enabled excellent water transport, salt rejection, and thermal insulation simultaneously in SGA, ultimately leading to highly efficient solar-powered water evaporation.

### Two-Way Water and Salt Transport Mechanism in SGA

Water and salt transport are critical to the evaporation performance and efficiency. The aerogel must effectively absorb and transport water upward to the water–air interface for sustained evaporation, while avoiding salt accumulation by rejecting salt ions downward [59]. The gradient pore channels of SGA endowed a unique two-way water and salt transport mechanism mimicking the water and sugar transport in the vascular system of trees, which is more effective than common aerogels with random or vertically aligned pore channels.

**Fig. 3:**
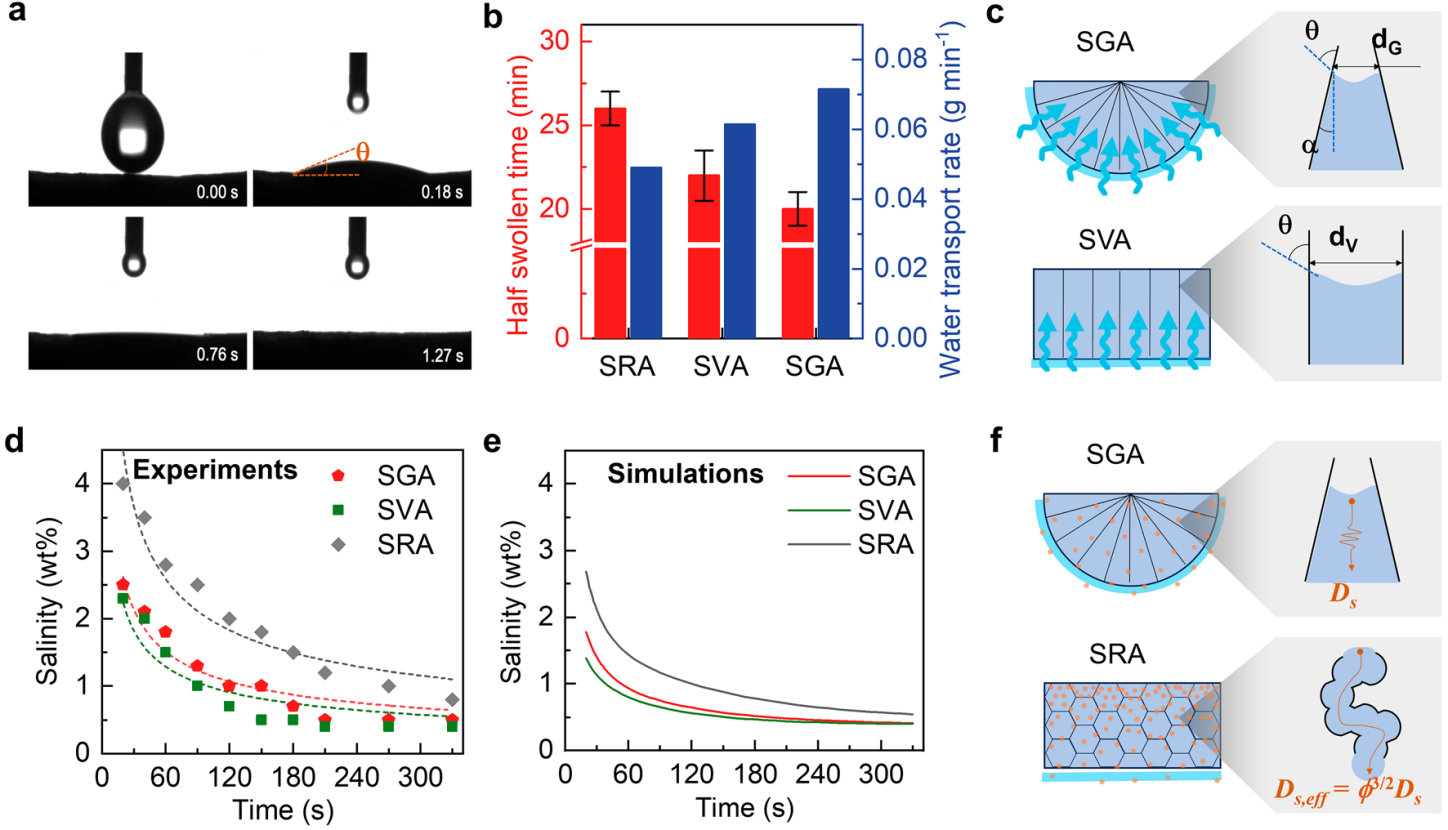
Two-way water and salt transport mechanism in SGA. **a** Time-dependent contact angles of water with the surface of SGA. **b** Half swollen time and water transport rates of three structures. **c** Schematics showing the mechanisms for better water transport in SGA than SVA because of a larger surface area for water absorption and a higher capillary pressure due to tapered pore channels in the former. **d** Experimental data (solid points) fitted with Fick's second law (dash lines) and **e** simulation results for surface salinity changes of three structures as a function of time after dripping 1 mL 20 wt% brine under the isothermal condition. **f** Schematics showing the mechanism for more effective downward salt transport of SGA than SRA because of the higher effective mass diffusivity due to direct transport pathways in the former

To evaluate the water transport capability, we first measured the contact angles of water on the evaporation surface and the bottom surface, as shown in Figs. 3a and S10, respectively. Both surfaces showed similar contact angles of 22° when the water droplet touched the surfaces, indicating a highly hydrophilic SGA because of PVA. Moreover, the water droplet was fully absorbed within only 1.27 s thanks to the large surface area arising from the fine-root-like secondary ligaments (Fig. 2d). Further, we investigated the water transport rate of SGA by measuring the half swollen time and saturated water content using Eq. 1 [60]:

1 $$v=\frac{0.5}{t}\left(\frac{{m}_{t}-{m}_{0}}{{m}_{0}}\right)$$

where *v* is the water transport rate, *m*_*t*_ and *m*_*0*_ are the masses of SGA in the initial and fully swollen states, respectively, and *t* is the time taken to full swelling. To highlight the advantage of the gradient pore channels in water transport, we also fabricated two other typical aerogels with random and vertical pore channels (Fig. S11), *i.e.*, structurally random aerogel (SRA) and structurally vertical aerogel (SVA), for comparison. It is noted that SRA and SVA were prepared using the same material composition and freezing temperature as SGA to maintain a uniform processing condition.As shown in Fig. 3b, the half swollen time of SGA was lower than both SRA and SVA, indicating a faster water uptake. Based on the mass changes and time, the water transport rate of SGA was 17 and 40% higher than SVA and SRA, respectively.

The best water transport properties of SGA among the three can be understood from the multiscale gradient structures, as schematically shown in Fig. 3c. Macroscopically, compared to common SRA and SVA with flat bottoms, the SGA could achieve a larger contact area for water absorption owing to the arched bottom and fan-shaped microstructures. Microscopically, the direct channels in SGA and SVA led to less resistance to water flow than the tortuous pathways in SRA, giving rise to higher water transport rates in the former. Furthermore, the water transport in SGA and SVA was driven by capillary pressures generated at the water–air interface, which were vastly different given their different pore geometries (enlarged view in Fig. 3c). The capillary pressure is given by the Young–Laplace equation:

2 $${\Delta p}_{G}=\frac{4\gamma \text{cos}(\theta -\alpha )}{{d}_{G}}$$

where *γ* is the surface tension of water, *θ* is the water contact angle with the channel wall, *α* is the angle between the cell wall and vertical direction, and *d*_*G*_ is the diameter of the pore channel at the meniscus. For SGA, a small *d*_*G*_ of 9 µm and a large *α* of 10° were observed from the SEM image (Fig. 2g) thanks to the tapered channels, generating a large ${\Delta p}_{G}$ for pumping the water to the evaporation surface. In comparison, the capillary pressure in SVA, ${\Delta p}_{v}$, took the same form as Eq. 2 but with *α*= 0° and a large diameter, *d*_*V*_, of  ~30 µm at the meniscus (Fig. S11a) because of the uniform diameter throughout the pore channel. Therefore, ${\Delta p}_{G}/{\Delta p}_{v}$ was calculated as 3.6, meaning 2.6 times larger capillary pressure was generated from SGA than SVA because of the gradient pore channel and smaller pore size at the meniscus of SGA. To verify the effect of pore size on water transport, we compared the water transport rates of two SVA samples with typical widths between cell walls of 10 µm (SVA-10) and 30 µm (SVA-30) prepared using different freezing temperatures of -120 and -80 ºC, respectively (Fig. S12a). SVA-10 showed a higher water transport rate than SVA-30 (Fig. S12b), further confirming the better water transport brought by narrower channels thanks to the higher capillary pressure. This larger capillary pressure was mainly responsible for the faster water transport in SGA than SVA with vertical channels.

In addition to upward water transport, the gradient channels in SGA also contributed to enhanced downward ion transport for salt rejection. The abilities of different structures to reject salt accumulation were compared under isothermal conditions. 1 mL brine with a concentration of 20 wt% was dripped onto the surface of SGA floating on DI water in 20 s. The salinity on the surface was measured using a refractometer (see Experimental Section for detailed salinity measurement) at different time intervals, as shown in Fig. 3d, which was compared to the other two structures. SGA and SVA showed more rapid decline in surface salinity than that of SRA, indicating faster salt transport in the former two structures, while negligible difference was noted between SGA and SVA in the salinity profiles. To understand the salt transport mechanism, the experimental data were first fitted using Fick’s second law (dash lines in Fig. 3d, see detailed fitting method in Supplementary Note S2). The fittings for the three structures agreed well with the experimental data, suggesting that the downward salt transport was mainly controlled by diffusion driven by the concentration gradient. To confirm the experimental finding, we further carried out numerical simulation using a time-dependent mass diffusion-convection model coupled with incompressible flow field (see detailed numerical method in Supplementary Note S3) [44]. As shown in Fig. 3e, the simulation result agreed well with the experimental finding that SGA and SVA showed faster decays in surface salt concentration than SRA, corroborating the better downward salt transport in SGA and SVA with aligned pore channels. The salinity distributions of three structures obtained from simulations are compared in Fig. S13. The salt distributions became almost uniform across the thickness of SGA and SVA after 120 s, while it took a longer time of 180 s for the SRA to reach equilibrium. The above experimental and simulation results substantiated the better downward salt transport performance of SGA and SVA than SRA by diffusion. The tortuous pathways of random pores in SRA generated large resistance to salt transport with a reduced effective mass diffusivity (Fig. 3f), *D*_*s,*eff_ = *ϕ*^3/2^*D*_*s*_, where *ϕ* is the porosity and *D*_*s*_ is the intrinsic diffusivity of salt ions in water [4]. By contrast, the aligned channels in SGA and SVA imposed much less resistance to ion transport, leading to an effective diffusivity close to the intrinsic value. Combined with the fast upward water transport driven by the capillary pressure to replenish the evaporation surface with sufficient water, the two-way water and salt transport mechanism of SGA was expected to avoid salt accumulation for long-term solar-driven evaporation.

### Simultaneous Heat Localization and Salt Rejection of SGA

In addition to fast water transport and salt rejection, effective heat localization is another critical factor contributing to a high evaporation rate and energy efficiency. However, there is often a tradeoff between excellent heat localization and salt rejection because of the highly coupled heat and mass transport in the porous evaporator [4, 44]. Although the analyses in the previous section demonstrate the effective salt rejection of SGA manifested by the unimpeded diffusion under the isothermal condition, it remains unclear if (i) simultaneous heat localization can be achieved and (ii) the diffusion can still be an effective way to reject salt when thermal effect is considered. Here, we first investigated the heat localization mechanism of SGA by quantifying different heat loss mechanisms and then probed into the salt rejection mechanism under coupled heat and mass transport.

Considering the energy balance of an evaporator, the power for evaporation (*Q*_evap_) comes from the absorbed power from the sun (*Q*_sun_) with some being lost to the surrounding (*Q*_loss_), namely

3 $${Q}_{\text{evap}}={Q}_{\text{sun}}-{Q}_{\text{loss}}$$

Given various energy loss mechanisms, the energy balance can be expressed as [4]:

4 $$\dot{m}{h}_{\text{LV}}=A\alpha {q}_{\text{solar}}-A\varepsilon \sigma \left({{T}_{s}}^{4}-{{T}_{\infty }}^{4}\right)-A{h}_{a}\left({T}_{s}-{T}_{\infty }\right)-\frac{A{k}_{\text{evap}}\left({T}_{s}-{T}_{\infty }\right)}{t}$$

The left-hand side of Eq. 4 is the power consumed for evaporation where $\dot{m}$ is the evaporation rate (in g s^−1^) and *h*_*LV*_ is the evaporation enthalpy of water (in J g^−1^). The first term on the right-hand side of Eq. 4 is the power absorbed from the sun where *A* is the top surface area of the evaporator, *α* is the solar absorption, and *q*_*solar*_ is the input solar power per area. The second term represents the power of radiation loss to the ambient where *ε* is the emissivity, *σ* is the Stefan-Boltzmann constant. The third term describes the power of convection loss to the ambient where *h*_*a*_ is the convective heat transfer coefficient of air. The last term corresponds to the conduction loss to bulk water where *k*_evap_ is the TC and *t* is the thickness of evaporators.

**Fig. 4:**
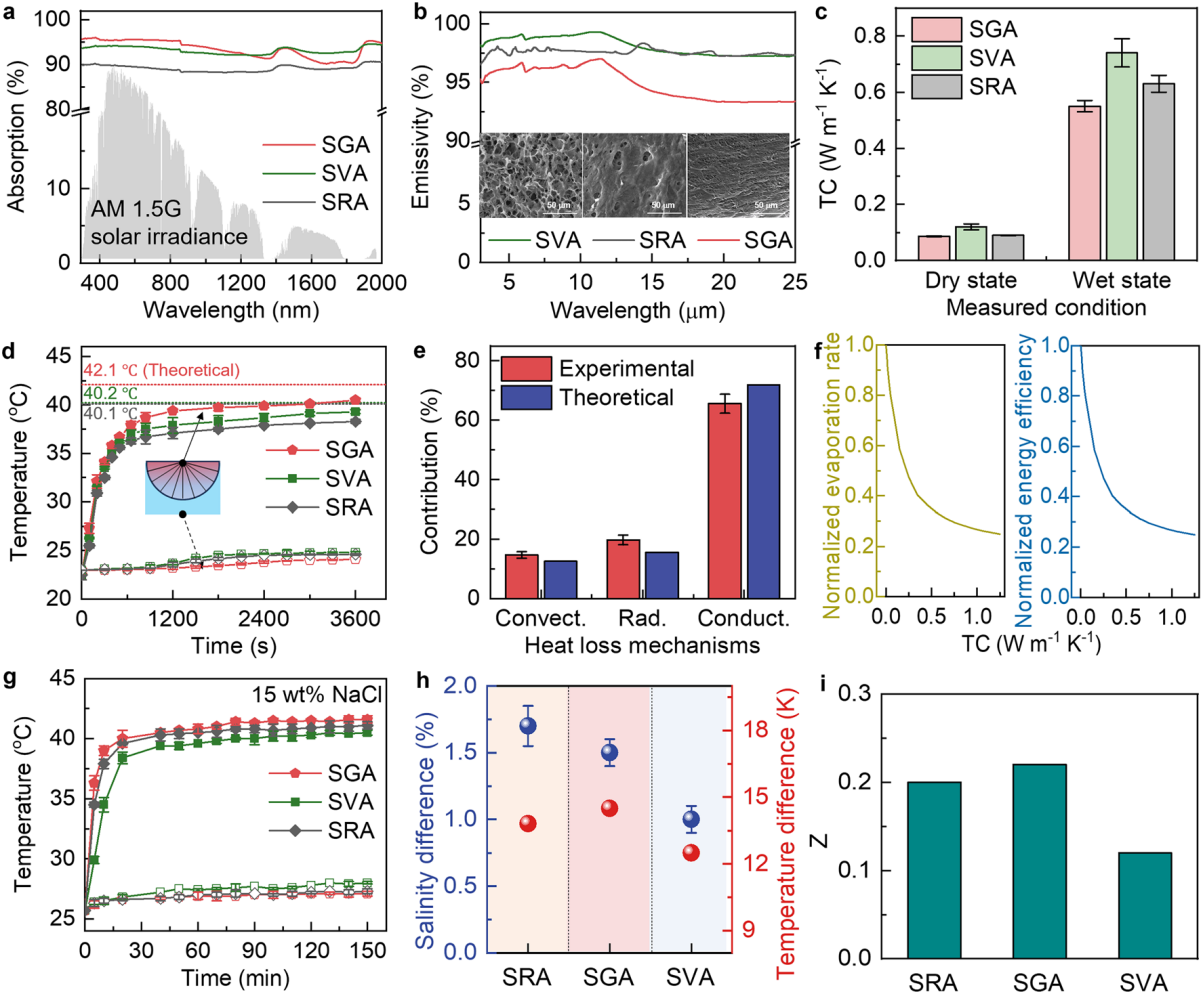
Simultaneous thermal localization and salt rejection of SGA. **a** Solar absorption spectra, **b** MIR emissivity, and **c** TCs of SRA, SVA, and SGA. **d** Temperature changes of the evaporator surface (solid data point) and bulk water (hollow data point) during evaporation tests in pure water under one-sun irradiation. The dashed lines are the theoretical surface temperatures predicted from the model. **e** Contributions of different heat loss mechanisms calculated from experiments and theoretical models. **f** Effect of TC on evaporation rate and energy efficiency. **g** Temperature changes of the evaporator surface (solid data point) and bulk water (hollow data point) during evaporation tests under one-sun illumination in 15 wt% NaCl brine. **h** Temperature and salinity difference between evaporation surface and bulk water in steady-state evaporation. **i** Figure of merit, *Z*, of three structures calculated from Eq. 6

According to the energy balance in Eq. 4 and assuming the same *h*_*a*_ for different structures, three thermal management strategies can be used to achieve a high evaporation rate, namely, maximizing the solar absorption (*α*), reducing the emissivity (*ε*), and minimizing TC (*k*_evap_). The solar absorption spectra of three different structures, namely SRA, SVA, and SGA, are shown in Fig. 4a. It is noted that the absorption was measured using samples in wet states by fully saturating the three samples with water before the measurements. The wet samples aligned better with the practical evaporation condition than the dry ones. Owing to the excellent photothermal properties of GO and CNTs [61, 62], the three structures all showed high solar-weighted absorption of over 85%. Among the three, SGA showed the highest solar absorption of 93% thanks to the flat top surface arising from the in-plane cell walls, resulting in a larger effective area for sunlight absorption than both SRA and SVA. The bottom surface of SGA showed similar solar absorption (Fig. S14) compared to the top surface. In addition, the flat top surface also contributed to a reduced emissivity of SGA despite the nearly 100% emissivity of GO and CNT fillers [63]. As shown in Fig. 4b, SVA and SRA showed a high emissivity of 98.5% and 97.1%, respectively, in the mid-infrared (MIR, 3–25 µm) band because of their highly porous surface (inset of Fig. 4b). By contrast, SGA showed a reduced emissivity of 95.7% thanks to its flat surface (insets of Fig. 4b), beneficial for the low radiative heat loss. The heat loss to bulk water was mainly determined by the thermal conduction through the aerogel, warranting a low TC to mitigate the conductive heat loss. The TCs of three aerogels measured in the thickness direction are shown in Fig. 4c. In the dry state, SVA showed the highest TC of 0.12 W m^−1^ K^−1^ because the vertically aligned cell walls conducted the heat effectively in the thickness direction (Fig. S11a). In comparison, SRA exhibited a lower TC of 0.09 W m^−1^ K^−1^ thanks to the tortuous conductive paths created by the random pore walls (Fig. S11b). The lowest TC of 0.085 W m^−1^ K^−1^ was achieved in SGA, which was 29% lower than that of SVA. After fully swelling the samples with water, SGA still maintained the lowest TC of 0.55 W m^−1^ K^−1^ among the three (Fig. 4c), even lower than that of water (~ 0.6 W m^−1^ K^−1^). The lower TC of swollen SGA than water could be attributed to the presence of pores in SGA even when saturated [48], which was attested by its low density even in a wet state (Fig. S15). The low TC of swollen SGA contributed to the inhibited heat conduction to bulk water. The better thermal insulation performance of SGA arose from the horizontal arrangement of pore channels close to the surface, which limited the heat transport in the transverse to alignment direction and thus achieving the best insulation in the thickness direction.

The above analyses indicate that the gradient porous structure of SGA improved solar absorption while mitigating various heat loss mechanisms better than its counterparts, essential for an excellent heat localization on the evaporation surface. To evaluate the heat localization in the three structures, we monitored the temperatures of both aerogel surface and bulk water at 2 cm beneath the aerogel during evaporation tests under one-sun irradiation, as shown in Fig. 4d. For SGA, a rapid rise in surface temperature from 23 to 36 °C within 10 min was observed (red solid data points in Fig. 4d). With the increasing irradiation time, the steady-state surface temperature of SGA reached 40.6 °C, 5.7% and 9.1% higher than those of SVA and SRA, respectively. Furthermore, the temperature of bulk water beneath SGA was only elevated by 1.1 °C (red hollow data points in Fig. 4d), which was the lowest among the three structures and thus corroborated the excellent thermal localization brought by the fan-shaped pore channels.

To verify the experimental findings, we used a theoretical model with coupled heat and vapor transport to quantitatively understand the dominant heat loss mechanism and its effect on the evaporation rate. In an open system where vapor diffuses from the evaporating surface to the ambient, the evaporation rate is determined by [64]:

5 $$\dot{m}={M}_{v}{D}_{v}S({c}_{\text{sat}}\left({T}_{s}\right)-{\phi }_{\infty }{c}_{\text{sat}}\left({T}_{\infty }\right))$$

where *M*_*v*_ is the molar mass of vapor (in g mol^−1^), *D*_*v*_ is the mass diffusivity of vapor in air (in m^2^ s^−1^),* S* is the shape factor relating to the vapor transport resistance due to evaporator geometry, *ɸ*_*∞*_ is the relative humidity at the far-field ambient, *T*_*s*_ and *T*_*∞*_ are the temperatures of the evaporator surface and the far-field ambient, respectively, and *c*_sat_(*T*_*s*_) and *c*_sat_(*T*_*∞*_) are the corresponding saturated vapor concentrations (in mol m^−3^). The surface temperatures of three structures in the steady state were calculated by solving Eqs. 4 and 5, shown as the dash lines in Fig. 4d (constants and parameters used in the calculation are listed in Table S1). The theoretical steady-state surface temperature of SGA is 42.1 °C, higher than that of SVA (40.2 °C) and SRA (40.1 °C), consistent with the experimental trends. To further understand the dominant heat loss mechanism in SGA, we calculated various heat loss components using the theoretical model and compared with those obtained from the experimental data, as shown in Fig. 4e (calculation details are provided in Supplementary Note S4 and Table S2). Both theoretical and experimental results suggest thermal conduction to bulk water was the major heat loss mechanism, contributing to ~ 70% of the total heat loss. Since thermal conduction dominated heat loss, the lower TC of SGA than SVA and SRA was the main reason for the better heat localization. Further, we probed the effect of TC of SGA on the evaporation rate and energy efficiency using the theoretical model. The evaporation rate and energy efficiency were normalized over the values when TC was 0 (*i.e.*, the aerogel was treated as an ideal thermal insulator, see Supplementary Note S5 for details), and the effects of TC on the normalized evaporation and energy efficiency are shown in Fig. 4f. Both the evaporation rate and energy efficiency decreased parabolically with the increasing TC. When the TC was increased to 0.15 W m^−1^ K^−1^, the evaporation rate and energy efficiency were only ~ 60% that of the ideally insulated case, indicating the importance of a low TC on achieving a high evaporation rate and energy efficiency.

After revealing the excellent thermal insulation of SGA and its dominating role in heat localization for high evaporation rate and energy efficiency, we moved on to probe the salt rejection capabilities of SGA under steady-state evaporation at one-sun irradiation. Although the salt rejection of SGA under the isothermal condition (*i.e.*, no solar radiation) has been demonstrated in Sect. 3.3, it is essential to confirm the simultaneous heat localization and salt rejection under practical evaporating conditions by considering coupled heat and salt transport. The SGA was floated on a 15 wt% NaCl solution with 0.5 g solid NaCl placed on its top surface initially (Fig. S16a). Under one-sun illumination, the salt particles dissolved gradually and completely disappeared after 110 min (Fig. S16b), indicating effective salt rejection by downward salt transport through aligned pore channels in SGA under sunlight. The salt rejection mechanism of SGA can be understood as follows. Because the dynamic balance between salt accumulation rate due to evaporation and the downward ion diffusion rate determines the salt crystal nucleation on the evaporation surface, it is desired to increase downward diffusion and upward water transport simultaneously for salt rejection [19]. On the one hand, the aligned microchannels in SGA facilitated salt ion transport, leading to higher downward ion diffusivity than SRA with a random porous structure. On the other hand, the tapered microchannels in SGA induced a higher capillary pressure than those of uniform width in SVA, giving rise to faster upward water transport in the former so that sufficient water was supplied to the evaporation surface for reduced salt concentration [65]. Therefore, the two-way water and salt transport mechanism enabled by the graded structure was responsible for the excellent salt rejection of SGA under solar irradiation.

To understand the mechanism behind simultaneous salt rejection and heat localization, we measured temperature difference, ∆*T*, and salinity difference, ∆*c*, between the top of evaporation surface and bulk water to quantitatively measure thermal and salt transport, respectively. The measurements were carried out under steady-state evaporation in 15 wt% high-concentration brine and one-sun irradiation. The temperatures of the evaporation surface and bulk water for the three structures were shown in Fig. 4g. The SGA showed a higher surface temperature than SVA and SRA, whereas SVA had the highest bulk water temperature. This observation is in line with the TCs of SGA and SVA, meaning that the downward salt transport did not alter the dominant heat loss mechanism (*i.e.*, thermal conduction). The temperature and salinity differences, ∆*T* and ∆*c*, between evaporation surface and bulk water of three structures are compared in Fig. 4h. In terms of thermal transport, SGA maintained the highest ∆*T* among the three, indicating the best heat localization on the evaporation surface. For salt transport, ∆*c* of SVA was the smallest, indicating the best salt transport because of the vertically aligned pores to maintain a small salinity difference. On the other hand, SRA showed the largest ∆*c* due to the random porous structure impeding the salt transport. The above analyses indicate that although SVA had a good salt rejection but lacked effective heat localization. Similarly, SRA showed decent thermal concentration while failing to reject salts. It is the fan-shaped gradient structure in SGA that enabled the simultaneous heat localization and salt rejection in the same evaporator. To quantify the material’s ability to simultaneously localize heat and reject salt, we propose a figure of merit, *Z*, as the ratio of the amount of salt rejected, *J*_salt_ to the conductive heat loss, *Q*_cond_ [66]:

6 $$Z=\frac{{J}_{\text{salt}}}{{Q}_{\text{cond}}}=\left[\frac{{D}_{s,\text{eff}}\rho A\left({c}_{s}-{c}_{\infty }\right)}{t}\right]/\left[\frac{A{k}_{\text{evap}}\left({T}_{s}-{T}_{\infty }\right)}{t}\right]=\frac{{D}_{s,\text{eff}}\cdot \rho \cdot \Delta c}{{k}_{\text{evap}}\cdot \Delta T}$$

where ${k}_{\text{evap}}$ is the TC of the evaporator, ${D}_{s,\text{eff}}$ is the effective diffusivity of salt in the evaporator, ρ is density of salt water, and *A* and *t* are the area and thickness of the evaporator. Essentially, *Z* represents the amount of salt rejected per joule of heat loss. As shown in Fig. 4i, SGA attained the highest *Z* value among the three structures, suggesting best salt rejection at the same heat loss. This is because the fan-shaped graded porous structure of SGA afforded both a high effective diffusivity (*D*_s,eff_) and a low TC (*k*_evap_), leading to a high *Z* value according to Eq. 6 for the simultaneous salt rejection and heat localization.

### Solar-Powered Water Evaporation Performance

**Fig. 5:**
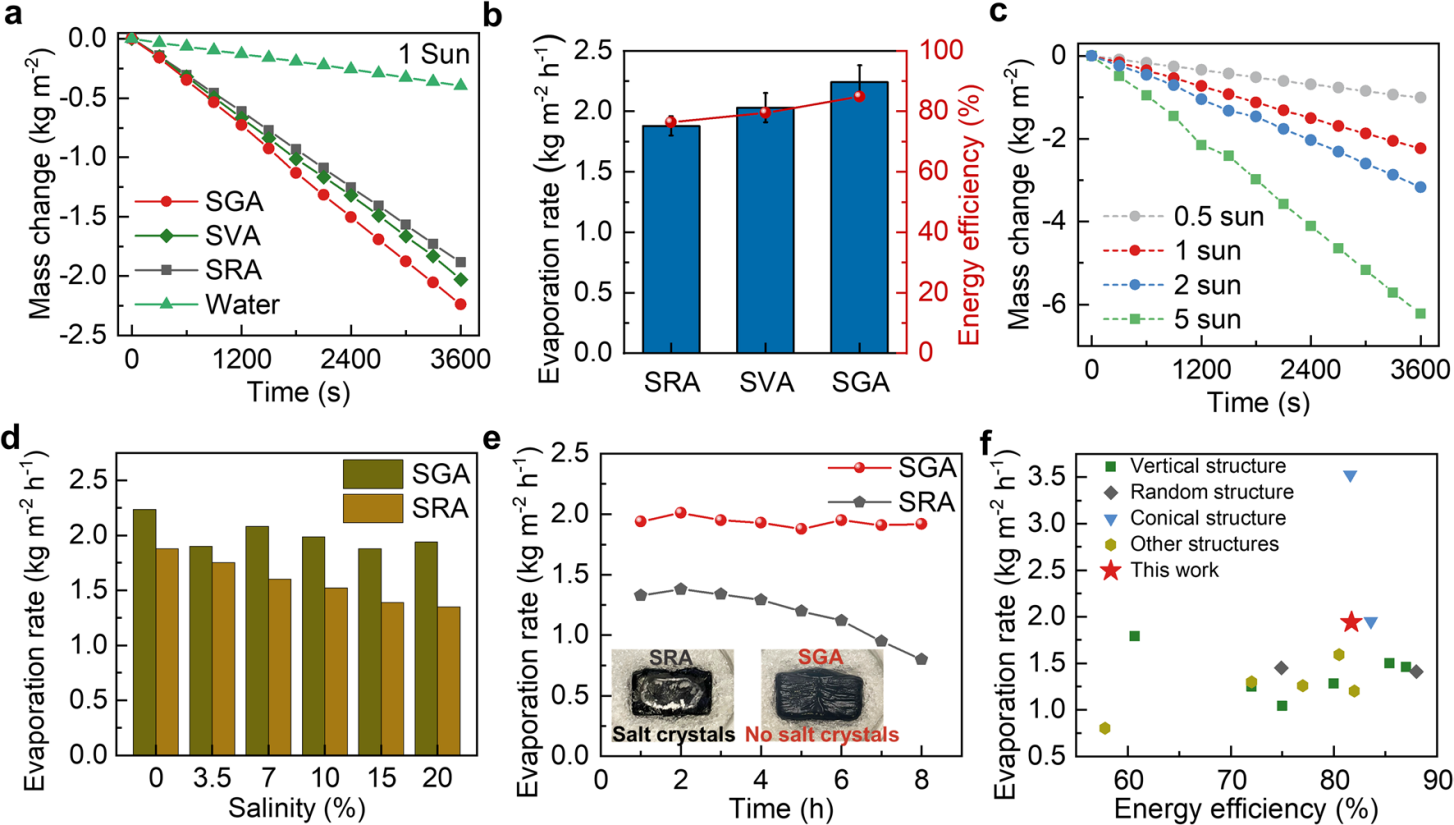
Evaporation performance of SGA. **a** Mass changes of water over time under one-sun illumination when different structures are used as evaporators. **b** Evaporation rates and energy efficiencies of SRA, SVA, and SGA. **c** Mass changes of SGA under different solar intensities. **d** Evaporation rates of SGA and SRA at different salinities. **e** The changes in evaporation rates of SRA and SGA in 20 wt% brine under one-sun illumination for 8 h. Insets show the salt accumulation on the surfaces of SRA after 8-h evaporation test while no salt accumulation observed for SGA.** f** Comparison of evaporation rate and energy efficiency under 1-sun achieved using SGA with other structural evaporators in 20 wt% NaCl solution [13, 33, 34, 36, 41, 59, 68–76]

The synergistically optimized water, salt and thermal transport make the SGA an excellent candidate in solar-powered water evaporation for stable desalination. The evaporation performance of SGA was systematically studied in DI water first and was compared with SRA and SVA. The three evaporators all showed lower densities than water (Fig. S15), enabling self-floating on the water surface. The mass changes of water for different aerogel evaporators under one-sun illumination are shown in Fig. 5a. The water evaporated at the fastest rate when SGA was used as the evaporator, reaching an evaporation rate of 2.24 kg m^−2^ h^−1^ (Fig. 5b), which was 5.6 times that of pure water. Under the same irradiation, the evaporation rates of SVA and SRA were 2.03 and 1.88 kg m^−2^ h^−1^, respectively, only 69.1% and 52.7% that of the SGA. To verify the potential of SGA for practical evaporation applications, evaporation rates were further measured at different solar intensities, as shown in Fig. 5c. The evaporation rate rose with the increasing light concentration and reached as high as 6.25 kg m^−2^ h^−1^ under 5-sun irradiation. It is worth noting that even under weaker irradiation (0.5 sun), the evaporation rate was still maintained at 1.01 kg m^−2^ h^−1^, making the aerogel suitable for practical desalination under weak sunlight. The solar-to-vapor conversion efficiencies of aerogels with different structures were calculated as:

7 $$\eta =\frac{\dot{m}{h}_{\text{LV}}}{A{q}_{\text{solar}}}$$

where $\dot{m}$ is the evaporation rate after subtracting dark evaporation, ${h}_{LV}$ is the equivalent evaporation enthalpy of water (Fig. S17), *A* is the top surface area of the evaporator, and *q*_*solar*_ is the input solar power. As shown in Fig. 5b, The SGA achieved an energy efficiency of 85% under one sun, consistently higher than SRA (76%) and SVA (80%). According to the water and thermal transport analyses in Sects. 3.3 and 3.4, the highest evaporation rate and energy efficiency of SGA among the three structures can be attributed to the faster water transport and lower conduction loss than both SGA and SVA, substantiating the advantage of fan-shaped gradient structure over conventional random and vertical structures in highly efficient water generation.

In addition to the evaporation rate, avoiding salt accumulation is another key to realize efficient and stable evaporation at a high salinity because salt accumulation could lower the evaporation rate or even incapacitating the evaporator. The effective salt rejection of SGA was made possible by the two-way water and salt transport mechanism (Sect. 3.3) without degrading the heat localization (Sect. 3.4). To verify the evaporation performance under high salinity, we measured the evaporation rates of SGA and SRA in NaCl solutions with salinities ranging from 0 to 20 wt% (Fig. 5d). The evaporation rate of SRA decreased continuously from 1.88 to 1.35 kg m^−2^ h^−1^ with the increasing salinity from 0 to 20 wt% because the random pores were not effective in rejecting salts at high salinities. By contrast, the evaporation rates of SGA in salt water were slightly reduced compared to that in pure water, but with no clear trend of deteriorating at high salinities. In fact, the evaporation rates in salt water remained relatively stable and reached 1.94 kg m^−2^ h^−1^ even in a 20 wt% NaCl solution, indicating a negligible effect of salts on the evaporation performance. The comparison clearly demonstrates the advantage of gradient pores to random ones in salt rejection for maintaining the evaporation performance at high salinities. To further verify the key role of the gradient structure in salt resistance, the evaporation performance of SGA was compared to SRA in a 20 wt% high-salinity brine, as shown in Fig. 5e. Unlike the consistent evaporation rate of SGA for 8 h, the evaporation rate of SRA began to decrease gradually after only 3 h of evaporation. After 8 h, the surface of SRA was completely covered by salt crystals (inset, Fig. 5e), leading to a significant drop in the evaporation rate to only 0.8 kg m^−2^ h^−1^. In contrast, the evaporation rate of SGA remained stable with no salt accumulation on the evaporation surface throughout the 8-h evaporation test. Even when the evaporation test was performed continuously for 36 h, the mass change remained linear with little variation in the evaporation rate during the whole test period (Fig. S18), demonstrating the long-term stability of SGA without salt accumulation under high-salinity conditions. The above comparison signifies the positive role of the fan-shaped gradient structure in achieving simultaneous heat localization and salt rejection to maintain the excellent evaporation performance at high salinities. Another possible factor affecting the evaporation rate and salt rejection performance is the pore channel size, which can be controlled by the freezing temperature [55]. We prepared SVA and SGA samples with different channel widths by using different freezing temperatures and compared their evaporation performance under one sun in a 20 wt% brine (see Supplementary Note S6 and Figs. S12, S19–S21 for details). We found that narrow pore channels exhibited fast evaporation at the initial stage thanks to the fast water transport due to high capillary pressures, but impeded the salt transport for deteriorated evaporation performance in the long run when salts accumulate on the evaporating surface (Figs. S19 and S21). Wide channels were preferred for avoiding salt accumulation, but they inevitably affected the heat localization, leading to low evaporation rate (Fig. S21). Therefore, a moderate channel width of ~ 30 µm was optimal for SGA for achieving a balance between salt rejection and heat localization.

The solar-powered evaporation performance of the SGA in the high-salinity NaCl solution (20 wt%) was compared with previously reported evaporators of different structures under the same condition (Fig. 5f). In a 20 wt% NaCl solution, the evaporation rate of SGA reached 1.94 kg m^−2^ h^−1^. This is much higher than those vertical and random structures reported in the literature. Although a conical-structured evaporator with a wide top and narrow bottom showed better performance (blue triangles in Fig. 5f) [49], it required additional support to float on water and the preparation material was complex, which greatly reduced its feasibility for practical application. The superior evaporation performance of SGA under high-salinity conditions was attributed to its unique graded structure with fan-shaped microchannels. Although a similar vertical radiant structure was reported previously for enhanced water transport, the vertical cell walls were not able to achieve proper thermal localization [57]. Similarly, bidirectionally aligned structures provided excellent thermal insulation [67], but the upward water transport and downward salt rejection were not optimized simultaneously. Compared to these similar structures reported, the graded structure of SGA realized fast upward water transport for evaporation and downward ion transport for salt rejection at the same time while also maintaining good thermal insulation.

### Practical Evaporation Performance and Stability

**Fig. 6:**
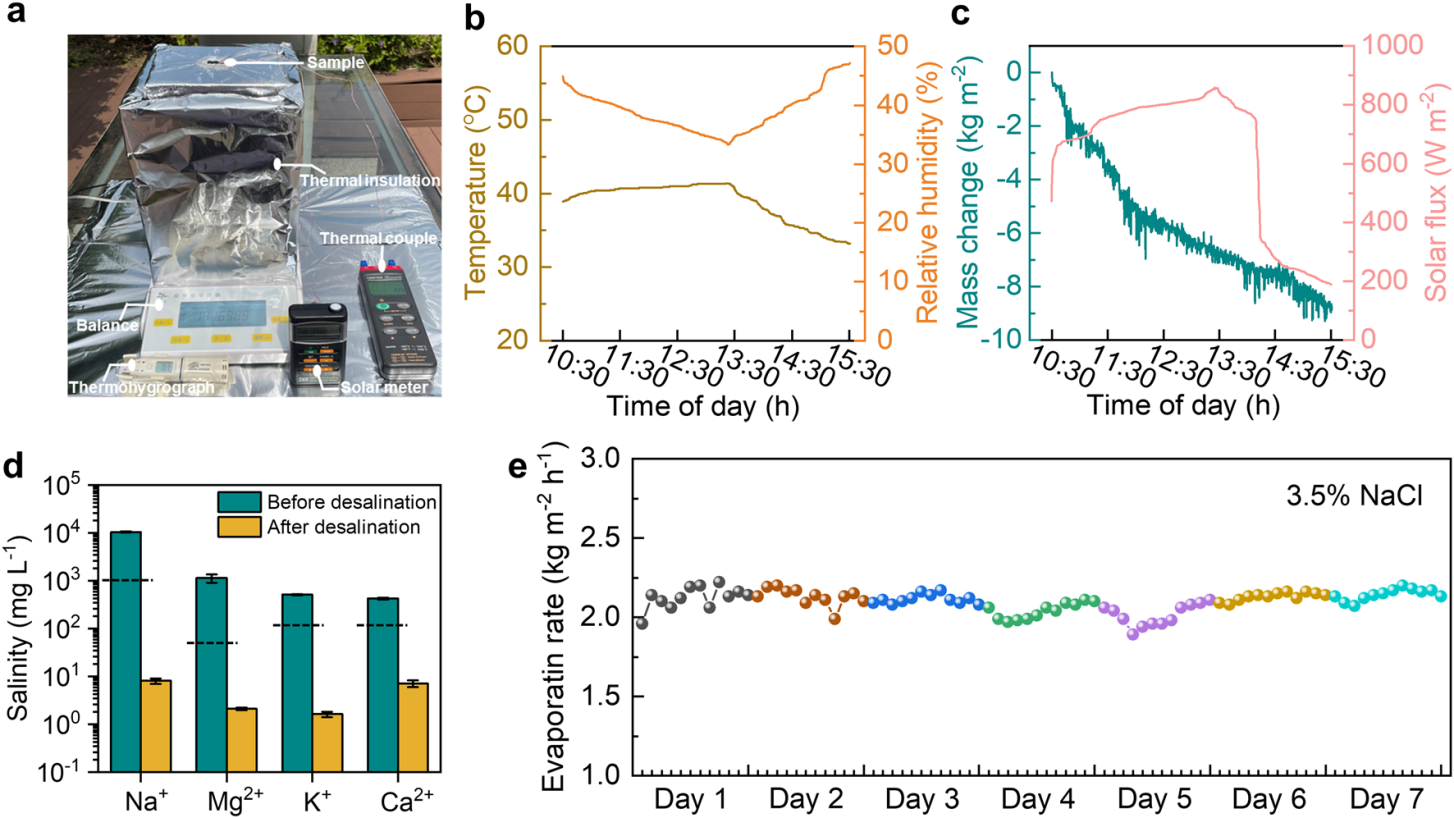
Demonstration of practical water desalination and long-term stability. **a** Photograph of the outdoor test setup. **b** Air temperature, relative humidity, **c** solar flux, and accumulated mass change of water from 10:30 to 15:30 during the outdoor evaporation test. **d** The concentrations of four major ions in water before and after desalination. Black dashed lines represent the thresholds for different ions recommended by the WHO for drinking water. **e** Long-term evaporation rates of SGA in a 3.5 wt% brine under one-sun illumination

The practical desalination application of SGA under natural sunlight was further investigated. SGA was placed in a beaker filled with seawater, which was enclosed in a custom-made desalination setup, as shown in Fig. 6a. On a typical sunny day in Hong Kong (3 June, 2023), the setup was exposed to sunlight for 5 h (10:30 to 15:30). The changes in ambient air temperature and relative humidity are shown in Fig. 6b. The solar intensity fluctuated during the testing period with an average irradiance of ~ 0.8 kW m^−2^, continuously producing fresh water through evaporation as evidenced by the mass loss in the beaker (Fig. 6c). The total amount of water evaporated during the 5-h period was 8.86 kg m^−2^, similar to the evaporation performance under the lab conditions. Moreover, the concentrations of four major ions (Na^+^, K^+^, Mg^2+^ and Ca^2+^) in the water were reduced by two to three orders of magnitude after evaporation, fully compliant with the World Health Organization (WHO) recommended drinking water standard (Fig. 6d). Furthermore, the solar absorption spectrum of SGA after the evaporation test was similar to that before the test with no apparent degradation, suggesting no loss of GO and CNTs to water or vapor during evaporation (Fig. S22).

In addition, the long-term desalination performance of SGA was also demonstrated in a NaCl solution with a salinity of 3.5 wt% similar to the seawater. The evaporation tests were carried out for 12 h under one sun followed by exposure in the dark for another 12 h to simulate the alternation of day and night, which lasted for 7 days in total. As shown in Fig. 6e, the evaporation rates fluctuated slightly from 1.89 to 2.22 kg m^−2^ h^−1^ during the 7-day measurements, averaging at 2.09 kg m^−2^ h^−1^. This value was very close to the one obtained in the evaporation test of pure water, demonstrating the stable operation of SGA in seawater-like brine for a prolonged period. A continuous evaporation test in the 3.5 wt% NaCl solution was also carried out for 240 h without interruption to confirm the long-term operation stability (Fig. S23). The evaporation rate remained stable during the whole testing period, averaging at 2.05 kg m^−2^ h^−1^. These results demonstrate the reliable long-term performance of SGA for practical solar-powered desalination and purification.

For large-scale applications, large areas of water need to be covered by SGA. Given the difficulty in producing SGA of larger sizes than a few centimeters limited by the freezing distance [46], we further demonstrated the use of multiple SGA floating on the water surface rather than a large piece for practical water production through solar-powered evaporation. As shown in Fig. S24a, 10 pieces of SGA covering the water surface with a total area of 20 cm^2^ were used for water-production demonstration in a custom-made water condensation device [77]. The solar desalination setup was designed to collect fresh water through water condensation and the water collection rate was evaluated in an outdoor test. Using a transparent plastic cap as the condenser, the condensed water was collected in a beaker at the bottom (Fig. S24b). The setup was exposed to natural sunlight for 7 h during the day and the corresponding solar intensity, ambient temperature, and relative humidity are shown in Fig. S24c. During the 7-h evaporation, the freshwater collection rate calculated based on the condensed water collected in the beaker reached 1.57 kg m^−2^  h^−1^, comparable to that of previous heat-localized evaporators [78]. The clear and transparent collected water after the evaporation test (Fig. S24d) also confirmed the stability of SGA without materials leaching [79]. Considering the importance of mechanical stability of SGA in practical applications, we measured the tensile and compressive properties of SGA (Fig. S25). The tensile stress–strain curve was measured using a hydrated sample, as shown in Fig. S25a. SGA exhibited a tensile strength of 0.33 MPa, much higher than recently reported hydrogel evaporators [80]. The compressive stress–strain curves of SGA aerogel are shown in Fig. S25b. The stress at 80% compressive strain of SGA was 8.08 MPa, which was much better than some common aerogels, such as covalent crosslinked polymer aerogels [81], MXene aerogels [82], and graphene aerogels [83]. The above analysis demonstrates the potential for large-scale application of SGA.

## Conclusions

In summary, inspired by the water and mass transport system of trees, a multiscale structurally graded GO-CNT/PVA aerogel was developed using a radial freeze-casting technique to achieve simultaneous fast water transport, excellent salt rejection, and heat localization for highly stable solar-powered evaporation under high-salinity condition. The two-way water and salt transport mechanism of SGA led to better water uptake and salt rejection than the vertical and random structures under isothermal conditions. In addition to fast water transport and salt rejection, effective heat localization under sunlight was simultaneously achieved in SGA by virtue of its planar surface and horizontal pore channels close to the surface. For the same amount of conductive heat loss, SGA demonstrated a larger amount of salt rejected than random and vertical structures, corroborating the unique advantage of the graded structure that allowed thermal localization and salt rejection to occur concurrently under high-salinity conditions. The synergistically fast water transport, excellent salt rejection, and heat localization empowered SGA impressive evaporation rates of 2.24 kg m^−2^ h^−1^ under one-sun illumination in DI water, which maintained stable at 1.94 kg m^−2^ h^−1^ in a 20 wt% NaCl solution without salt accumulation. Under the typical salinity of seawater, the evaporation rates also sustained consistently, averaging at 2.09 kg m^−2^ h^−1^ for 7 days without degradation. The integrated design of water, salt, and thermal transport as well as the simple preparation method developed in this work provides an enticing solution to solar-powered desalination under high-salinity conditions.

## Supplementary Information

Below is the link to the electronic supplementary material.

Supplementary file1 (DOCX 26727 kb)
