# Supplementary material for: Tree-Inspired Structurally Graded Aerogel with Synergistic Water, Salt, and Thermal Transport for High-Salinity Solar-Powered Evaporation
Source: Nanomicro Lett. 2024 Jun 17;16:222. doi: 10.1007/s40820-024-01448-8 (PMC11183023; doi:10.1007/s40820-024-01448-8)
Supplement: Supplementary file 1 — Supplementary file1 (DOCX 26727 kb) [file 40820_2024_1448_MOESM1_ESM.docx]

Supporting Information for

**Tree-Inspired Structurally Graded Aerogel with Synergistic Water, Salt, and Thermal Transport for High-Salinity Solar-Powered Evaporation**

Xiaomeng Zhao^1^, Heng Zhang^1^, Kit-Ying Chan^1,3^, Xinyue Huang^1^, Yunfei Yang^1^, Xi Shen^1,2,3,^*

^1^ Department of Aeronautical and Aviation Engineering, The Hong Kong Polytechnic University, Hung Hom, Kowloon, Hong Kong SAR, P. R. China

^2^ Research Institute for Sports Science and Technology, The Hong Kong Polytechnic University, Hung Hom, Kowloon, Hong Kong SAR, P. R. China

^3^ Research Institute for Advanced Manufacturing, The Hong Kong Polytechnic University, Hung Hom, Kowloon, Hong Kong SAR, P. R. China

*Corresponding author. E-mail: [xi.shen@polyu.edu.hk](mailto:xi.shen@polyu.edu.hk) (Xi Shen)

**Supplementary Note S1 Optimization of GO, CNT and PVA Concentrations**

**﻿**A higher carbon nanofiller (GO and CNT) concentration in the aerogel can lead to better solar absorption, but inevitably reduce porosity which is undesirable for water uptake. Therefore, the weight ratio of carbon nanofillers to PVA was optimized to achieve a balance for a high evaporation rate. The SGA samples with different GO-CNT:PVA weight ratios of 0.025, 0.035, 0.05 and 0.075, designating as GO-CNT/PVA-0.025, GO-CNT/PVA-0.035, GO-CNT/PVA-0.05 and GO-CNT/PVA-0.075, respectively, were tested under one sun. The weight ratio of GO and CNT were kept the same at 3:7 for all compositions. The corresponding evaporation rates were 1.34, 1.71, 2.24, and 2.13 kg m^-2^ h^-1^, respectively (Fig. S4). The evaporation rate first increased to 2.24 kg m^-2^ h^-1^ at an optimal weight ratio of 0.05:1 before turning to decline. The difference in evaporation rate was attributed to the microstructure change caused by the change of GO-CNT/PVA ratio. As shown in Fig. S5, with the increasing GO-CNT:PVA ratio, the pores became increasingly smaller with less obvisouly pore alignment. Although the samples with different GO-CNT:PVA ratios were produced under the same freezing temperature (-80 °C) using the same mold, the lack of alignment at a high GO-CNT:PVA ratio of 0.075 was mainly due to the high viscosity of the solution, resisting the ice crystals to grow unidirectionally during freeze-casting and thus forming rather random pore structures. This means that the porosity for water transport was reduced with the increasing filler concentration, which caused the evaporation rate to decrease when the filler-to-PVA ratio exceeded 0.05:1.

The weight ratio of PVA/water in the solution was also optimized. The SGA samples with PVA/water weight ratios of 0.07, 0.10, and 0.12, designated as PVA/water-0.07, PVA/water-0.10, and PVA/water-0.12, respectively, were measured under one-sun illumination and the corresponding evaporation rates were shown in Fig. S6a. SGA with PVA/water-0.10 showed the highest evaporation rate of 2.24 kg m^-2^ h^-1^. Although increasing PVA concentration could increase the hydrophilicity for water uptake, it also led to the decrease of porosity and micropore size (Fig. S6b), in turn reducing the water transport through the aerogel network. Therefore, an optimal evaporative performance was achieved when the water uptake and porosity were in balance at the PVA/water weight ratio of 0.10.

**Supplementary Note S2 Fitting Model of Salt Transport**

The experimental data of surface salinity for three structures were fitted using the Fick’s second law:

$\frac{\partial c(x, t)}{\partial t}=D_{s,eff}\frac{\partial^{2}c(x,t)}{\partial x^{2}}$  (S1)

where *c(x, t)* is the concentration of salt and *D_s,eff_* is the effective diffusion coefficient. Assuming the total amount of salt dripped into the water is constant, the solution to Equation S1 is

$c\left( x,t \right)= \frac{Q_{0}}{\sqrt{\pi D_{s,eff}t}}\exp\left( -\frac{x^{2}}{4D_{s,eff}t} \right)$ (S2)

where *Q_0_* is the total amount of salt in the solution introduced on the sample surface.

**Supplementary Note S3 Numerical Model of Salt Transport**

The numerical modeling of salt transport was carried out using COMSOL Multiphysics software. Based on the geometry of three structures, a two-dimensional computing domain was constructed for SGA, SVA, and SRA, respectively (Fig. S13). To simulate the brine dripping process, a layer of 20 wt% brine was uniformly formed on the surface of the sample as the initial condition. The incompressible flow field was described by [S1]:

$\rho\frac{\partial\boldsymbol{u}}{\partial t}+\rho\left( \boldsymbol{u}\cdot\nabla\right) \boldsymbol{u}=-\nabla p+\nabla\cdot\left[ \mu(\nabla\boldsymbol{u}+{(\nabla\boldsymbol{u})}^{T} \right]+\rho\boldsymbol{g}$ (S3)

$\nabla\cdot\boldsymbol{u}=0$ (S4)

where $\rho$, $\boldsymbol{u}$, $p$, $\mu$, $T$, and $\boldsymbol{g}$ are the mass density, vector flow field, pressure, viscosity, temperature and gravitational acceleration, respectively.

The salt transport was determined using a time-dependent mass diffusion-convection model:

$\frac{\partial c}{\partial t}-\nabla\cdot(D_{s}\nabla c)+\boldsymbol{u}\cdot\nabla c=0$ (S5)

where $c$ is the concentration of the species and $D_{s}$ is the diffusion coefficient.

**Supplementary Note S4 Heat Loss Analysis**

The total heat loss power ($Q_{loss}$) during evaporation consists of three parts, namely, radiation, convection, and conduction:

$Q_{loss}=Q_{rad}+Q_{conv}+Q_{cond}$ (S6)

where the three terms at the right-hand side represent the power of radiation loss, convection loss, and conduction loss, respectively.

The heat loss contribution is calculated as the ratio of different heat loss components to the total heat loss. The detailed calculation of each heat loss component is provided in the following.

**(1) Radiation loss**

The radiation loss is calculated by

$Q_{rad}=A\varepsilon\sigma\left( {T_{s}}^{4}-{T_{\infty}}^{4} \right)$ (S7)

where $A$ is the surface area (2 cm^2^), 𝜀 is the emissivity of SGA (95.7%), 𝜎 is the Stefan-Boltzmann constant (5.67×10^-8^ W m^-2^ K^-4^), $T_{s}$ is the surface temperature of SGA (~40.5 °C), and $T_{\infty}$ is the ambient temperature (~37.6 °C) measured by a thermocouple placed above the evaporation surface. For the calculation of radiation loss from theoretical model, $T_{s}$ (42.1 °C) and $T_{\infty}$ (22.5 °C) were the theoretical temperatures obtained by solving Equations (4) and (5).

**(2) Convection loss**

The convection loss is calculated by:

$Q_{conv}=Ah_{a}\left( T_{s}-T_{\infty} \right)$ (S8)

where $h_{a}$ is the convection heat transfer coefficient (~ 5 W m^-2^ K^-1^ for air under natural convection). *T_s_* and *T_∞_* were the same as those used in calculating the radiation loss.

**(3) Conduction loss**

During the experiment, the conduction loss was calculated according to the temperature of the underlying bulk water as follows:

$Q_{cond}=Cm\Delta T$  (S9)

﻿where $C$ is the specific heat capacity of water (4.2 J g^-1^ °C^-1^), $m$ is the mass of bulk water (10 g), $\Delta T$ is the change in underlying bulk water temperature before and after evaporation (~ 1.1 °C).

For the theoretical model, the conduction loss is calculated as

$Q_{cond}=\frac{T_{s}-T_{\infty}}{\frac{1}{Sk_{w}}+\frac{t}{Ak_{evap}}}$ (S10)

where $k_{w}$ is the TC of water (0.6 W m^-1^ K^-1^), $S$ is the shape factor relating to the vapor transport resistance due to evaporator geometry (0.02 m), $k_{evap}$ is the TC of evaporator (0.55 W m^-1^ K^-1^) and $t$ is the thickness of evaporator (0.01 m).

**Supplementary Note S5 Effect of TC on Evaporation Rate and Energy Efficiency**

The dominant role of thermal conduction in determining the heat loss and its effect on the evaporation rate and energy efficiency were evaluated based on the theoretical model given by Equations 4 and 5. Different *k_evap_* values were used as the inputs to obtain the surface temperature of SGA under steady state by solving Equations 4 and 5, and then the corresponding evaporation rates at different *k_evap_* were calculated using Equation 5. The evaporation rates were then normalized over that at *k_evap_* = 0 (i.e., no conduction loss) and plotted against *k_evap_* in Fig. 4f.

The energy efficiency is calculated as

$\eta^{'}=\frac{Q_{evap}}{Q_{sun}}=1-\frac{Q_{loss}}{Q_{sun}}$ (S11)

where Q_sun_ is the input solar power and Q_loss_ is calculated according to Supplementary Note S4. Similar to evaporation rate, energy efficiencies at different *k_evap_* were calculated and normalized over that at at *k_evap_* = 0 (i.e., no conduction loss). The normalized energy efficiency is plotted against *k_evap_* in Fig. 4f.

**Supplementary Note S6 Effect of Pore Channel Size on the Evaporation Rate and Salt Rejection**

To understand the effect of pore channel size on the evaporation performance, we first prepared SVA samples with two typical widths between cell walls (Fig. S12a), 10 µm (SVA-10) and 30 µm (SVA-30) by using different freezing temperatures of -120 and -80 °C, respectively. The evaporation performance of SVA-10 and SVA-30 in a 20 wt% brine for 8 hours under one sun was shown in Fig. S19. The evaporation rate of SVA-10 in the initial evaporation stage was higher than that of SVA-30 as indicated by the faster water loss, which was attributed to the higher water transport rate of the former. However, as evaporation continued, salt precipitation was clearly observed on the evaporation surface of SVA-10 (inset of Fig. S19), resulting in gradually reduced evaporation rates. By contrast, SVA-30 showed consistent evaporation rate in the 8-hour test without salt accumulation (inset of Fig. S19). The better salt rejection performance of SVA-30 than SVA-10 was attributed to the wider channel width in the former which facilitate salt transport, giving rise to consistent evaporation performance in the long run. The above analysis indicates that although a wider channel width was inferior to the narrow one in the initial stage of evaporation due to the smaller capillary pressure, it performed more consistently than its narrow counterpart under extended period of evaporation because of the better salt rejection.

We also prepared SGA samples with different channel widths and compared their evaporation performance. Three SGA samples with different channel widths of 10 µm (SGA-10), 30 µm (SGA-30), and 50 µm (SGA-50) were prepared using different freezing temperatures of – 120, – 80, and – 30 ºC, respectively (Fig. S20). It is noted that the channel width here refers to the width between cell walls at the bottom of SGA. The evaporation performance was evaluated under one sun for 8 hours in a 20 wt% brine, as shown in Fig. S21. Compared to SGA-30, SGA-10 showed a higher evaporation rate of 2.21 kg m^-2^ h^-1^ at the initial 3 hours of evaporation, but the rate gradually decreased with the increasing operation time, primarily due to salt accumulation on the evaporation surface with narrow channel width in SGA-10. On the other hand, a large channel width in SGA-50 contributed to a stable evaporation for the entire 8 hours, but its excessively wide channels adversely affected the thermal localization, in turn resulting in a low evaporation rate of only 1.6 kg m^-2^ h^-1^. By contrast, the moderate channel width of ~30 µm in SGA-30 balanced the salt rejection and heat localization, giving rise to a stably high evaporation rate ~ 1.94 kg m^-2^ h^-1^ for 8 hours without salt accumulation.

In summary, the above results elucidate the effect of channel width on evaporation rate and salt rejection. Narrow channels induced high capillary pressure for faster evaporation at the initial stage but impeded the salt transport under high-salinity conditions in the long run for deteriorated evaporation performance due to salt accumulation. Wide channels were preferred for avoiding salt accumulation, but they inevitably affected the heat localization, leading to low evaporation rate. Therefore, a moderate channel width was essential to balance the salt rejection and heat localization. In our case, a channel width of ~30 µm was found to be optimal and was used for SGA.

**Supplementary Figures**


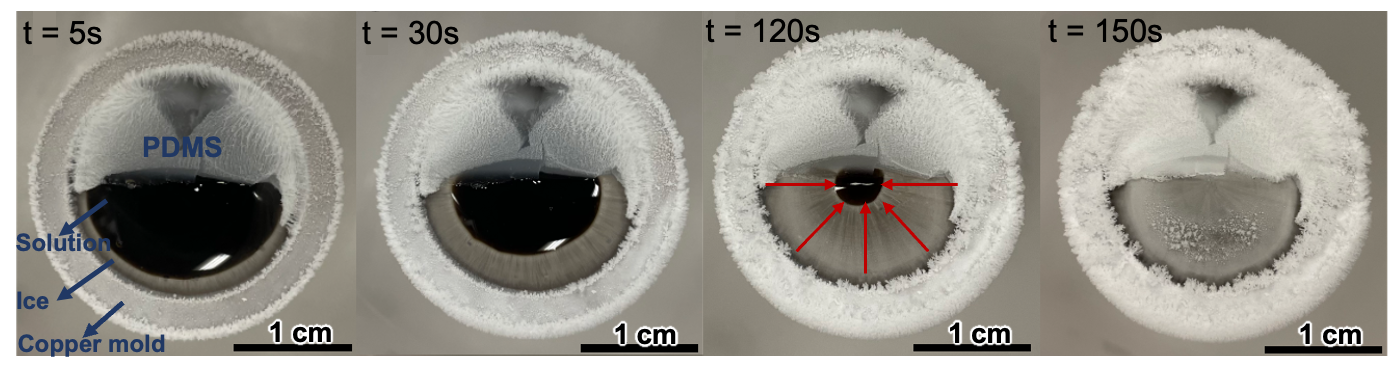


**Fig. S1** Photographs showing the radial growth of ice crystals over time

**Fig. S2 a** SGA of different sizes (top) and their applications for practical water production by placing multiple SGAs on the water surface (bottom). **b** Demonstration of buoyancy stability for SGAs with two different sizes, i.e., one of 1.0 by 2.5 cm and the other of 2.0 by 3.5 cm


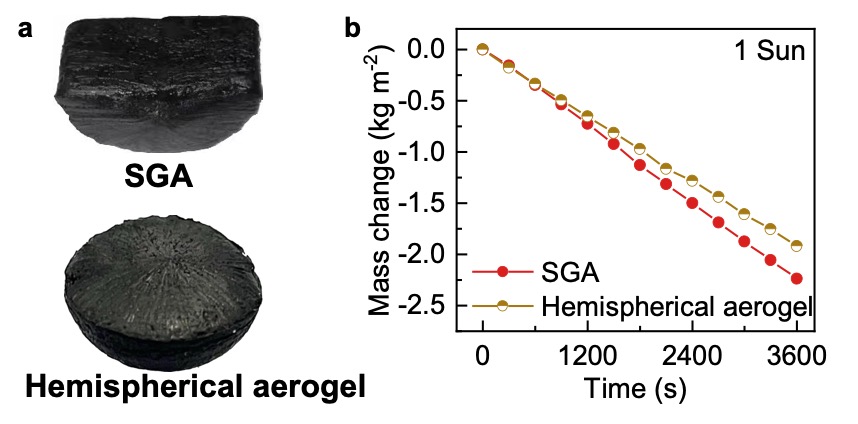


**Fig. S3 a** Photographs of SGA and hemispherical aerogel. **b** Evaporation performance of SGA and hemispherical aerogel


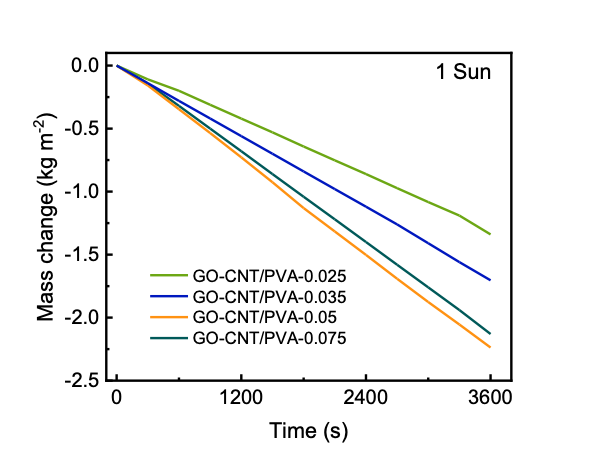


**Fig. S4** The mass changes of water during evaporation tests under one-sun illumination for SGA samples with different GO-CNT/PVA ratios


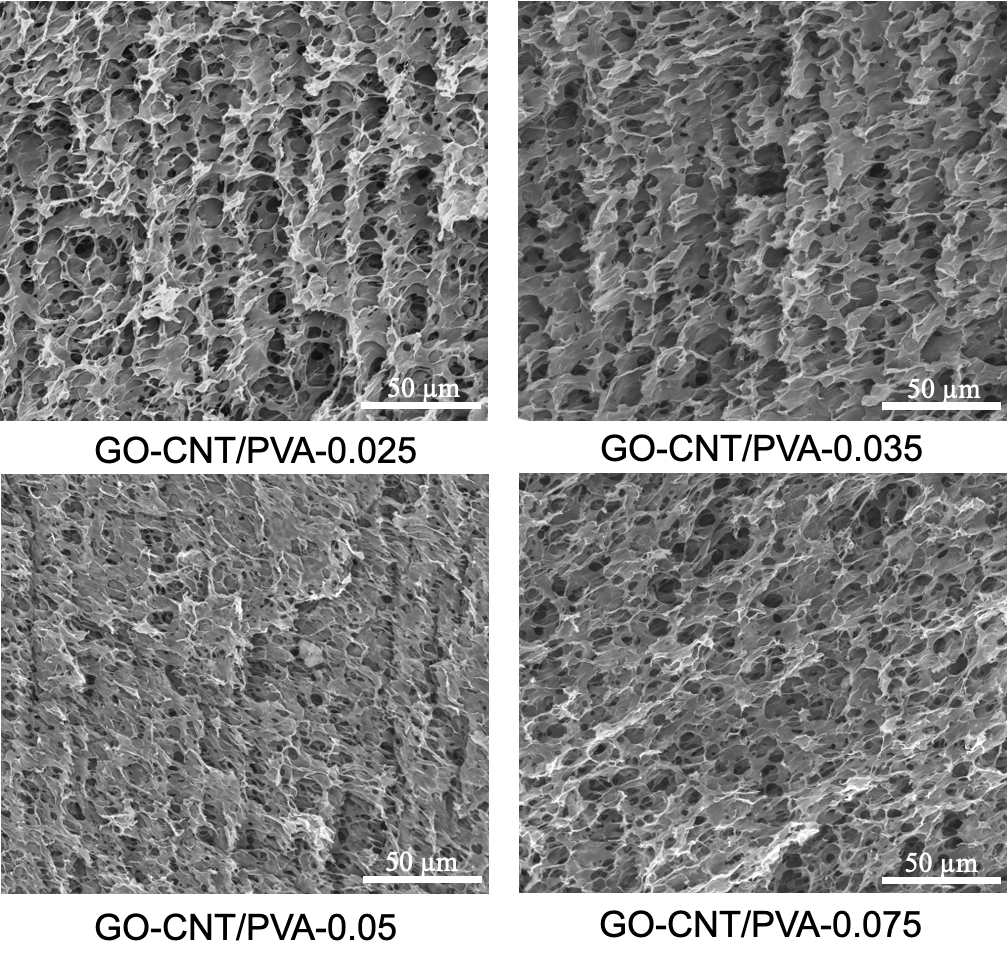


**Fig. S5** SEM images of SGA samples with different GO-CNT/PVA ratios


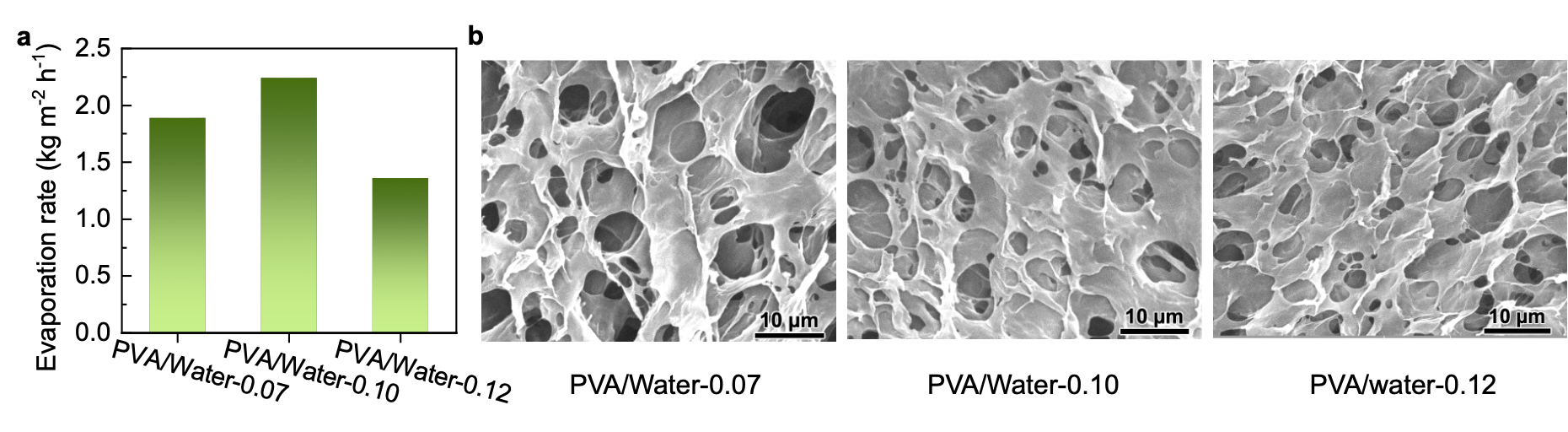


**Fig. S6** **a** The evaporation rates and **b** SEM images of SGA samples with different PVA/water ratios

**Fig. S7** Raman spectra of GO, CNT, GO-CNT mixture, PVA powder and SGA. GO, CNT and GO-CNT mixture exhibited two characteristic peaks at 1348 and 1587 cm^-1^ corresponding to the D and G bands [S2]. For the pure PVA, a prominent peak at 2904 cm^-1^ was assigned to the stretching vibrations of -CH_2_ [S3]. The Raman spectra of SGA contained characteristic peaks of both GO-CNT and PVA, indicating the presence of GO, CNT, and PVA in SGA

**Fig. S8** Demonstration of buoyant stability of **a** SGA with a hull-like, arched bottom and **b** aerogel with a rectangular shape. After a large displacement was applied, SGA was able to oscillate and eventually return to its stable position because the lower center of bouncy than the center of gravity. By contrast, the aerogel with a rectangular shape was flipped over by the same displacement, demonstrating the improved stability of the hull-like structure


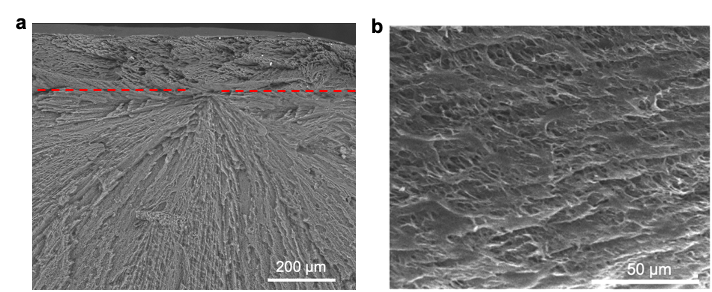


**Fig. S9 a** The cross-sectional SEM image of SGA showing the horizontal cell walls on the surface. **b** The surface SEM image of SGA showing the presence of micro-sized pores

**Fig. S10** The water contact angle on the bottom surface of the SGA


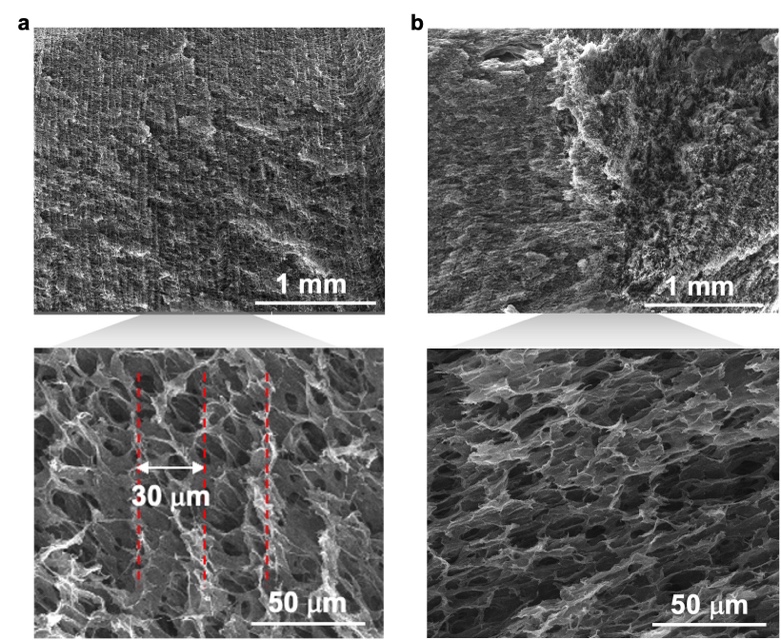


**Fig. S11** Cross-sectional SEM images of **a** SVA and **b** SRA at different magnifications

**Fig. S12 a** SEM images and **b** water transport rates of SVA-10 and SVA-30


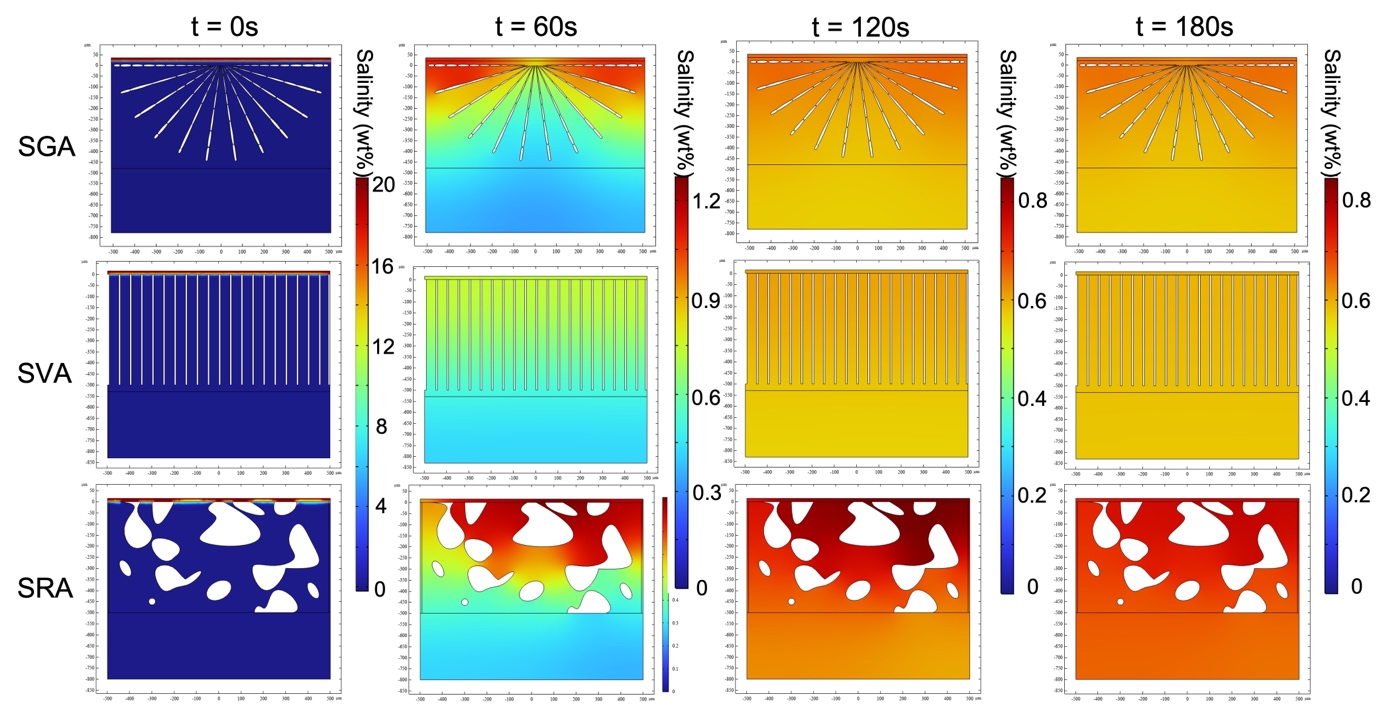


**Fig. S13** The salinity distributions of three structures after dripping 1 mL 20 wt% brine under an isothermal condition

**Fig. S14** Solar absorption spectrum of the bottom surface of SGA

**Fig. S15** The densities of SRA, SVA and SGA in dry and wet states


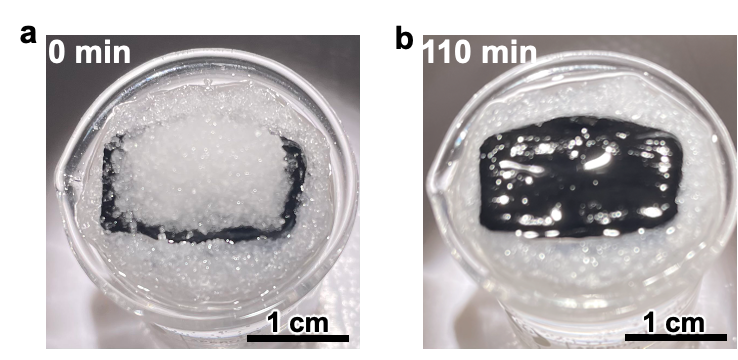


**Fig. S16** Photographs showing **a** 0.5g NaCl placed on the surface of SGA before evaporation test and **b** complete dissolving of NaCl after 110 min of water evaporation under one-sun illumination


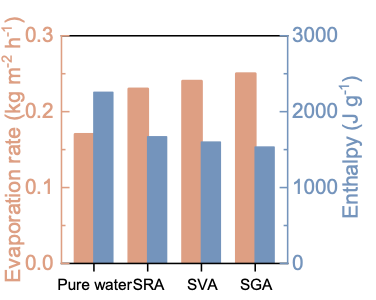


**Fig. S17** The evaporation rate under the dark condition and the equivalent enthalpy of pure water, SRA, SVA and SGA

The dark evaporation tests were carried out using different samples and pure water with the same surface area. The sample were put into a container at room temperature and atmospheric pressure without illumination [S4]. Mass changes of SGA, SVA, SRA and pure water were measured using an electronic balance to obtain their dark evaporation rates. The equivalent enthalpy $h_{LV}$ of water in SGA, SVA and SRA was calculated by [S5]

$h_{w}m_{0}=h_{LV}m_{g}$ (S12)

where $h_{w}$is the evaporation enthalpy and $m_{0}$ is the mass change of pure water; $h_{LV}$ and $m_{g}$ are the equivalent evaporation enthalpy and mass changes of SGA, SVA and SRA. Compared to pure water, the aerogels exhibited lower evaporation enthalpy and higher dark evaporation rates. This is because the hydroxyl groups in the PVA networks could form strong hydrogen bonds with the water molecules, weakening the hydrogen bonds between the water molecules and causing the water molecules to escape more easily into the air [S6].

**Fig. S18** The mass change of SGA in a 20 wt% NaCl solution under one-sun illumination for 36 h

**Fig. S19** The mass changes of SVA with different pore channel widths in a 20 wt% brine under one-sun illumination for 8 h. Insets show the salt accumulation on the surfaces of SVA-10 during the test while no salt accumulation observed for SVA-30

**Fig. S20** SEM images showing channel widths of SGA-10, SGA-30, and SGA-50 freeze-cast at – 120, – 80, and – 30 ºC, respectively. Note that SGA-30 was the optimal one and designated as SGA in the main text

**Fig. S21** The changes in evaporation rates of three SGA samples in a 20 wt% brine under one-sun illumination for 8 h

**Fig. S22** Solar absorption spectra of SGA before and after evaporation test

**Fig. S23** Long-term evaporation rates of SGA in a 3.5 wt% brine under one-sun illumination for 240 hours without interruption

**Fig. S24 a** Photographs and dimensions of the custom-made water condensation device. An aluminum foil was used to cover the water surface between evaporators to avoid evaporation directly from water. **b** Photograph of the solar desalination setup. **c** The solar flux, temperature, and humidity during the test. **d** Photograph of collected fresh water in the beaker

**Fig. S25 a** Tensile stress-strain curve and **b** compressive stress-strain curves of SGA

**Supplementary Tables**

**Table S1** Various constants and parameters used in the calculation of theoretical surface temperatures of SGA, SVA and SRA

| **Constants** | | | | |
| --- | --- | --- | --- | --- |
| **Symbol** | **Description** | **Value** | | |
| $\sigma$ | Stefan-Boltzmann constant [W m^-2^ K^-4^] | 5.67×10^-8^ | | |
| $D_{v}$ | Diffusivity of vapor in air [m^2^ s^-1^] | 3×10^-5^ | | |
| $M_{v}$ | Molar mass of air [kg mol^-1^] | 18.015×10^-3^ | | |
| $h_{a}$ | Convective heat transfer coefficient to ambient [W m^-2^ K^-1^] | 5 | | |
| $q_{solar}$ | Solar flux [W m^-2^] | 1000 | | |
| $T_{\infty}$ | Far field ambient temperature [K] | 295.65 | | |
| $\phi_{\infty}$ | Far field relative humidity | 1 | | |
| **Parameters** | | | | |
| **Symbol** | **Description** | **SGA** | **SVA** | **SRA** |
| *L* | Length [m] | 0.01 | 0.01 | 0.01 |
| A | Area of evaporator [m^2^] | 2×10^-4^ | 2×10^-4^ | 2×10^-4^ |
| $t$ | Thickness [m] | 0.01 | 0.01 | 0.01 |
| $\alpha$ | Solar absorptance | 0.93 | 0.92 | 0.89 |
| $\varepsilon$ | Solar emissivity | 0.957 | 0.985 | 0.971 |
| $h_{lv}$ | Enthalpy of vaporization [J kg^-1^] | 1534×10^3^ | 1598×10^3^ | 1667×10^3^ |
| $k_{evap}$ | Effective TC of evaporator [W m^-1^ K^-1^] | 0.55 | 0.74 | 0.63 |
| $S$ | Shape factor [m] | A/L | A/L | A/L |

**Table S2** The heat loss contributions of SGA calculated from theoretical model and experimental data

|  | **Radiation loss (%)** | **Convection loss (%)** | **Conduction loss (%)** |
| --- | --- | --- | --- |
| Theoretical model | 15.52 | 12.54 | 71.94 |
| Experimental data | 19.71 | 14.71 | 65.58 |

**Table S3** Comparison of evaporation performance under 1-sun in 20 wt% NaCl solution

| **Structure** | **Materials** | **Evaporation rate (kg m^-2^ h^-1^)** | **Energy efficiency (%)** | **Refs** |
| --- | --- | --- | --- | --- |
| Vertical structure | ﻿ CNTs/SiO_2_ | 1.5 | 85.4 | [S7] |
|  | Attapulgite/cellulose/PVA/PPy | 1.28 | 80 | [S8] |
|  | ﻿ PVA/polyacrylamide/MXene | 1.79 | 60.7 | [S9] |
|  | MXene | 1.46 | 87 | [S10] |
|  | Silica nanofibers | 1.25 | 72 | [S11] |
|  | Carbonized wood | 1.04 | 75 | [S12] |
| Random structure | ﻿Polymer foam/PPy | 1.41 | 88 | [S13] |
|  | Carbonized loofah | 1.45 | 74.9 | [S14] |
| Conical structure | PVA/MXene fibre cloth | 1.95 | 83.6 | [S15] |
|  | SPI/HEC/epichlorohydrin/ CB | 3.53 | 81.6 | [S4] |
| Other structures | Resin/carbon nanofibers | 1.59 | 80.5 | [S16] |
|  | ﻿Basswood/graphitic carbon | 1.3 | 72 | [S17] |
|  | ﻿PDA/PEI/PPy/PI | 1.26 | 77 | [S18] |
|  | Resin/carbon nanoparticles | ~ 0.8 | 57.8 | [S19] |
|  | Janus wood | 1.2 | 82 | [S20] |
| **Graded structure** | **GO-CNT/PVA** | **1.94** | **81.7** | **This work** |

**Supplementary References**

1. L. Zhang, X. Li, Y. Zhong, A. Leroy, Z. Xu, et al. Highly Efficient and Salt Rejecting Solar Evaporation *via* a Wick-Free Confined Water Layer. Nat. Commun. **13**(1), 849 (2022). <https://doi.org/10.1038/s41467-022-28457-8>
2. D. Koo, J. Sung, H. Suh, S. Bae, and H. So. Comprehensive Analysis of CNT/NS/GO Composites: Dispersion Effect of Graphene Oxide for Environmental Sensor Application. Compos. Part A Appl. Sci. Manuf. **173**, 107639 (2023). <https://doi.org/10.1016/j.compositesa.2023.107639>
3. Y. Shi, D. Xiong, J. Li, K. Wang, and N. Wang. *In situ* Repair of Graphene Defects and Enhancement of Its Reinforcement Effect in Polyvinyl Alcohol Hydrogels. RSC Adv. **7**(2), 1045-1055 (2017). <https://doi.org/10.1039/c6ra24949c>
4. X. Liu, F. Chen, Y. Li, H. Jiang, D.D. Mishra, et al. 3D Hydrogel Evaporator with Vertical Radiant Vessels Breaking the Trade-Off between Thermal Localization and Salt Resistance for Solar Desalination of High-Salinity. Adv. Mater. **34**(36), e2203137 (2022). <https://doi.org/10.1002/adma.202203137>
5. H. Zhang, X. Shen, E. Kim, M. Wang, J.H. Lee, et al. Integrated Water and Thermal Managements in Bioinspired Hierarchical MXene Aerogels for Highly Efficient Solar-Powered Water Evaporation. Adv. Funct. Mater. **32**(19), 2111794 (2022). <https://doi.org/10.1002/adfm.202111794>
6. Z. Yu and P. Wu. Biomimetic MXene-Polyvinyl Alcohol Composite Hydrogel with Vertically Aligned Channels for Highly Efficient Solar Steam Generation. Adv. Mater. Technol. **5**(6), 2000065 (2020). <https://doi.org/10.1002/admt.202000065>
7. X. Dong, L. Cao, Y. Si, B. Ding, and H. Deng. Cellular Structured CNTs@SiO_2_ Nanofibrous Aerogels with Vertically Aligned Vessels for Salt-Resistant Solar Desalination. Adv. Mater. **32**(34), e1908269 (2020). <https://doi.org/10.1002/adma.201908269>
8. L. Song, L. Geng, Y. Tian, P. Mu, and J. Li. Robust Superhydrophilic Attapulgite-Based Aligned Aerogels for Highly Efficient and Stable Solar Steam Generation in Harsh Environments. J. Mater. Chem. A **9**(40), 23117-23126 (2021). <https://doi.org/10.1039/d1ta07042h>
9. 9. Z.Y. Wang, Y.J. Zhu, Y.Q. Chen, H.P. Yu, and Z.C. Xiong. Bioinspired Aerogel with Vertically Ordered Channels and Low Water Evaporation Enthalpy for High-Efficiency Salt- Rejecting Solar Seawater Desalination and Wastewater Purification. Small **19**(19), e2206917 (2023). <https://doi.org/10.1002/smll.202206917>
10. Q. Zhang, G. Yi, Z. Fu, H. Yu, S. Chen, et al. Vertically Aligned Janus MXene-Based Aerogels for Solar Desalination with High Efficiency and Salt Resistance. ACS Nano. **13**(11), 13196-13207 (2019). <https://doi.org/10.1021/acsnano.9b06180>
11. X. Dong, Y. Si, C. Chen, B. Ding, and H. Deng. Reed Leaves Inspired Silica Nanofibrous Aerogels with Parallel-Arranged Vessels for Salt-Resistant Solar Desalination. ACS Nano **15**(7), 12256-12266 (2021). <https://doi.org/10.1021/acsnano.1c04035>
12. Y. Kuang, C. Chen, S. He, E.M. Hitz, Y. Wang, et al. A High-Performance Self-Regenerating Solar Evaporator for Continuous Water Desalination. Adv. Mater. **31**(23), e1900498 (2019). <https://doi.org/10.1002/adma.201900498>
13. J. He, Z. Zhang, C. Xiao, F. Liu, H. Sun, et al. High-Performance Salt-Rejecting and Cost-Effective Superhydrophilic Porous Monolithic Polymer Foam for Solar Steam Generation. ACS Appl. Mater. Interfaces **12**(14), 16308-16318 (2020). <https://doi.org/10.1021/acsami.9b22832>
14. X. Wang, L. Zhang, D. Zheng, X. Xu, B. Bai, et al. A Polyelectrolyte Hydrogel Coated Loofah Sponge Evaporator Based on Donnan Effect for Highly Efficient Solar-Driven Desalination. Chem. Eng. J. **462**, 142265 (2023). <https://doi.org/10.1016/j.cej.2023.142265>
15. L. Li, N. He, B. Jiang, K. Yu, Q. Zhang, et al. Highly Salt-Resistant 3D Hydrogel Evaporator for Continuous Solar Desalination *via* Localized Crystallization. Adv. Funct. Mater. **31**(43), 2104380 (2021). <https://doi.org/10.1002/adfm.202104380>
16. M. Zou, Y. Zhang, Z. Cai, C. Li, Z. Sun, et al. 3D Printing a Biomimetic Bridge-Arch Solar Evaporator for Eliminating Salt Accumulation with Desalination and Agricultural Applications. Adv. Mater. **33**(34), e2102443 (2021). <https://doi.org/10.1002/adma.202102443>
17. H. Jang, J. Choi, H. Lee, and S. Jeon. Corrugated Wood Fabricated Using Laser-Induced Graphitization for Salt-Resistant Solar Steam Generation. ACS Appl. Mater. Interfaces. **12**(27), 30320-30327 (2020). <https://doi.org/10.1021/acsami.0c05138>
18. Z. Chen, Y. Luo, Q. Li, and X. Chen. Microgroove-Structured PDA/PEI/PPy@PI-MS Photothermal Aerogel with a Multilevel Water Transport Network for Highly Salt-Rejecting Solar-Driven Interfacial Evaporation. ACS Appl. Mater. Interfaces. **13**(34), 40531-40542 (2021). <https://doi.org/10.1021/acsami.1c09155>
19. Z. Wang, Z. Zhan, Y. Li, M. Xie, H. Kong, et al. A Tree-Root Mimicked Janus Evaporator for Solar Evaporation of Saturated Saline Water. J. Mater. Chem. A. **11**(48), 26592-26601 (2023). <https://doi.org/10.1039/d3ta05753d>
20. X. Chen, S. He, M.F. Mark, Y. Wang, T. Li, et al. Sustainable Off-Grid Desalination of Hypersaline Waters by Janus Wood Evaporator. Energy Environ. Sci. **14**(10), 5347-5357 (2021). <https://doi.org/https://doi.org/10.1039/D1EE01505B>
